# Supplementary material for: Proximity to small-scale inland and coastal fisheries is associated with improved income and food security
Source: Commun Earth Environ. 2022 Aug 3;3(1):174. doi: 10.1038/s43247-022-00496-5 (PMC9362682; doi:10.1038/s43247-022-00496-5)
Supplement: Supplementary file 1 — Supplementary Information [file 43247_2022_496_MOESM1_ESM.pdf]

Supplementary Information	
Supplementary Table 1	Survey overview and sample size
Supplementary Table 1a	Estimated probability to be income poor (living below the national poverty line) by quintile of distance to water bodies .
Supplementary Table 1b	Estimated marginal effects on the probability to be food insecure (households with below adequate food consumption scores), by quintile of distance to water bodies
Supplementary Table 2	Summary (unweighted) statistics for the econometric analysis
Supplementary Table 3a	Fish and other animal-source food consumption by proximity to water bodies
Supplementary Table 3b	Fish and other animal-source food consumption by proximity to water bodies - by poor/non-poor HHs (above and below national poverty lines)
Supplementary Table 3c	Fish and other animal-source food consumption by proximity to water bodies - by dietary diversity score (food consumption score)
Supplementary Table 4a	Fish consumption - form (dried/fresh) and source (purchased/own production/other) - by proximity to water bodies
Supplementary Table 4b	Fish consumption - form (dried/fresh) and source (purchased/own production/other) - by proximity to water bodies and poor/non-poor HHs (above and below national poverty lines)
Supplementary Table 5	Fish consumption - quantity - by quintile and proximity to water bodies
Supplementary Table 6_Malawi	Fishing HHs vs non-fishing HHs - fish consumption, dietary diversity and multiple poverty indicators
Supplementary Table 6_Tanzania	Fishing HHs vs non-fishing HHs - fish consumption, dietary diversity and multiple poverty indicators
Supplementary Table 6_Uganda	Fishing HHs vs non-fishing HHs - fish consumption, dietary diversity and multiple poverty indicators
Supplementary Table 6_All countries	Fishing HHs vs non-fishing HHs - fish consumption, dietary diversity and multiple poverty indicators
Supplementary Table 7	Dietary diversity (food consumption score) by proximity to water bodies
Supplementary Table 8	Multiple poverty indicators by proximity to water bodies
Supplementary Table 9	Estimated fish consumption - in KG / per capita / year - compared to FAO Supply Data, and scenario for increasing 1% fish supply
Supplementary Table 10	Prices of animal source food purchased by households
Supplementary Table 11	Variable description
Supplementary Table 12	Nutrient content of fish species caught from small-scale fisheries in the region and other animal source foods (per 100g edible food).
Supplementary Figure 1	Share of households consuming Animal source food and corresponding prices per Kg
Supplementary Figure 2	Map of HHs and fish consumption, fishing and non-fishing HHs
Supplementary Figure 3 & 4	Quantities and ratio of fish consumption (kg/household/week) between richest and poorest households by rural and urban areas and proximity to water bodies. Bars indicate the confidence interval in the calculated statistics at 95 % significant level.
Supplementary Figure 5	Fish consumption of rural households by proximity to water bodies
Supplementary Figure 6	Fig 5. Fish consumption (average kg/household/week) by food consumption profile and proximity to water bodies. Note: food consumption profile is based on the household Food Consumption Score (FCS), which measures frequency and diversity of food groups con
Supplementary Figure 7	Research framework - food systems

Supplementary Table 1 Survey overview and sample size

Unweighted statistics (Sample)

Country	Survey Name	Survey Year	Non-agriculture households	Fishing households	Agriculture Households	Total	% of non-agriculture households	% of fishing households	% of agriculture households
Malawi	Fourth Integrated Household Survey	2016-2017	2,096	414	9,937	12,447	16.8%	3.3%	79.8%
Tanzania	National Panel Survey, Wave 4	2014-2015	1,115	136	2,101	3,352	33.3%	4.1%	62.7%
Uganda	National Panel Survey	2010-2011	594	76	2,246	2,916	20.4%	2.6%	77.0%
Total			3,805	626	14,284	18,715	20.3%	3.3%	76.3%

Weighted statistics (using probability sampling weights)

Country	Survey Name	Survey Year	Non-agriculture households	Fishing households	Agriculture Households	Total	% of non-agriculture households	% of fishing households	% of agriculture households
Malawi	Fourth Integrated Household Survey	2016-2017	661,378	88,865	3,047,071	3,797,313	17.4%	2.3%	80.2%
Tanzania	National Panel Survey, Wave 4	2014-2015	2,559,630	429,097	7,161,940	10,150,667	25.2%	4.2%	70.6%
Uganda	National Panel Survey	2010-2011	1,325,739	133,244	3,669,974	5,128,957	25.8%	2.6%	71.6%
Total			4,546,746	651,205	13,878,985	19,076,937	23.8%	3.4%	72.8%

Overview of the LSMS surveys used in the study in Sub-Saharan Africa

Country	Survey Name	Survey Year	Total Sample Households[1]	Type of fishing livelihood activities	Number of Fishing Households	Survey livelihood question	Time of year data collected
Malawi	Fourth Integrated Household Survey	2016-2017	12,447	Harvesting, processing and trade	414	“Did any HH member engage in fishing or fish trading”	Throughout the year
Tanzania	National Panel Survey, Wave 4	2014-2015	3,352	Harvesting, processing and trade	136	"During the last 12 months, how many of these months did you, or someone in your household, engage in fishing, fish trading, or fish processing activities?"	Throughout the year
Uganda	National Panel Survey	2010-2011	2,916	Harvesting only	76	“Did anybody in the household practice A: river fishing; B: Natural freshwater pond/lake fishing; C Artificial fishpond fishing; D) swamp fishing”	Throughout the year
Total			18,715		626		

1 Sample size corresponds to the total number of households sampled in the survey. Due to missing predictors or outliers, the final number of households including in the probit regression are slightly less than the total number of households included in the descriptive statistics (18,610 households in the probit regression (see Table 1 and 2)).

Supplementary Table 1a Estimated probability to be income poor (living below the national poverty line) by quintile of distance to water bodies .

	**Poverty**															
	**All countries**				**Malawi**				**Tanzania**				**Uganda**			
	**National**		**Rural**		**National**		**Rural**		**National**		**Rural**		**National**		**Rural**	
**Quintile of distance to water bodies**	**Prob. to be poor**	**Dist.**	**Prob. to be poor**	**Dist.**	**Prob. to be poor**	**Dist.**	**Prob. to be poor**	**Dist.**	**Prob. to be poor**	**Dist.**	**Prob. to be poor**	**Dist.**	**Prob. to be poor**	**Dist.**	**Prob. to be poor**	**Dist.**
	*100	Km	*100	Km	*100	Km	*100	Km	*100	Km	*100	Km	*100	Km	*100	Km
**1**	**0.339**	**2.7**	**0.454**	**3.3**	**0.448**	**4.0**	**0.465**	**3.1**	**0.371**	**2.2**	**0.589**	**3.4**	**0.193**	**3.5**	**0.218**	**3.6**
2	0.288	11.8	0.431	15.7	0.439	20.4	0.536	18.2	0.351	10.5	0.563	17.1	0.121	10.8	0.170	13.1
3	0.373	26.3	0.449	31.3	0.395	35.8	0.546	36.6	0.443	25.5	0.588	32.9	0.206	20.5	0.232	25.9
4	0.427	44.7	0.486	48.3	0.490	50.7	0.537	52.6	0.506	45.5	0.559	50.2	0.233	37.7	0.250	41.7
5	0.491	79.3	0.571	80.9	0.458	74.3	0.501	75.2	0.544	85.9	0.668	88.6	0.303	68.5	0.326	71.6
**Mean**	**0.384**	**33.1**	**0.478**	**36.0**	**0.446**	**37.0**	**0.517**	**37.1**	**0.443**	**33.8**	**0.593**	**38.0**	**0.212**	**28.5**	**0.239**	**31.2**
Obs.	18,623		14,283		12,444		10,174		3,344		1,978		2,822		2,125	

Note: For each household, the estimated probability to be income poor is calculated as: $\Pr(Poor_i = 1) = \Phi(\beta X_i) + e_i$, where Φ is the cumulative normal distribution, X_iis the data vector consisting of observable characteristics for the i-th household, including the distance to water bodies, and β is the vector of coefficient estimates reported in Table 1A. Finally, e_i is the model error term.

Supplementary Table 1b Estimated probability to be food insecure (households with below adequate food consumption scores) by quintile of distance to water bodies.

	Food insecurity															
	All countries				Malawi				Tanzania				Uganda			
	National		Rural		National		Rural		National		Rural		National		Rural	
Quintile of distance to water bodies	Prob. to be food insecure	Dist.	Prob. to be food insecure	Dist.	Prob. to be food insecure	Dist.	Prob. to be food insecure	Dist.	Prob. to be food insecure	Dist.	Prob. to be food insecure	Dist.	Prob. to be food insecure	Dist.	Prob. to be food insecure	Dist.
	*100	Km	*100	Km	*100	Km	*100	Km	*100	Km	*100	Km	*100	Km	*100	Km
**1**	**0.206**	**2.7**	**0.268**	**3.3**	**0.609**	**4.0**	**0.643**	**3.1**	**0.124**	**2.2**	**0.156**	**3.4**	**0.151**	**3.5**	**0.155**	**3.6**
2	0.216	11.8	0.279	15.7	0.533	20.4	0.654	18.2	0.184	10.5	0.219	17.1	0.165	10.8	0.143	13.1
3	0.282	26.3	0.299	31.3	0.479	35.8	0.636	36.6	0.210	25.5	0.254	32.9	0.163	20.5	0.167	25.9
4	0.311	44.7	0.340	48.3	0.577	50.7	0.627	52.6	0.256	45.5	0.287	50.3	0.167	37.7	0.163	41.7
5	0.332	79.3	0.391	80.9	0.550	74.3	0.604	75.2	0.277	85.9	0.326	88.6	0.223	68.5	0.255	71.6
**Mean**	**0.270**	**33.1**	**0.315**	**36.0**	**0.549**	**37.0**	**0.633**	**37.1**	**0.210**	**33.8**	**0.210**	**38.0**	**0.174**	**28.5**	**0.177**	**31.2**
Obs.	18,623		14,283		12,444		10,174		3,344		1,971		2,822		2,125	

Note: For each household, the estimated probability to be food insecure was calculated as: $\Pr(Poor_i = 1) = \Phi(\beta X_i) + e_i$, where Φ is the cumulative normal distribution, X_i is the data vector consisting of observable characteristics for the i-th household, including the distance to water bodies, and β is the vector of coefficient estimates reported in Table 1B. Finally, e_i is the model error term.

Supplementary Table 2 Summary (unweighted) statistics for the econometric analysis

Main variables used for the econometric analysis (unweighted statistics)									
		Malawi		Tanzania		Uganda		All countries	
VARIABLES		mean	sd	mean	sd	mean	sd	mean	sd
Indicators	Measure								
Poor household	% of total household	0.447	0.497	0.426	0.495	0.217	0.412	0.408	0.491
Food consumption profile									
Poor	1 poor; 0 otherwise	0.143	0.35	0.0418	0.2	0.0432	0.203	0.109	0.312
Borderline	1 borderline; 0 otherwise	0.385	0.487	0.147	0.354	0.124	0.33	0.302	0.459
Acceptable	1 acceptable; 0 otherwise	0.472	0.499	0.811	0.391	0.832	0.374	0.589	0.492
Rural households	% of total households	0.817	0.386	0.592	0.492	0.746	0.436	0.766	0.423
Age of the head of the household	Age	43.29	16.19	44.42	14.99	45.11	15.22	43.77	15.84
Sex of the head of the household	1 male; 0 female	0.713	0.452	0.715	0.451	0.719	0.45	0.714	0.452
Household size	number of household members	4.329	2.001	4.858	2.849	6.322	3.327	4.734	2.519
Education of the head of the household									
Primary education	1 primary education; 0 otherwi	0.679	0.467	0.232	0.422	0.24	0.427	0.531	0.499
Secondary education	1 Secondary education; 0 other	0.199	0.399	0.553	0.497	0.524	0.5	0.313	0.464
Tertiary education	1 Tertiary education; 0 otherwi	0.104	0.305	0.199	0.4	0.178	0.382	0.133	0.339
Not stated		0.0177	0.132	0.0155	0.124	0.058	0.234	0.0235	0.152
Distance to nearest agriculture markets	Km	22.45	17.26			29.32	20.14	23.76	18.04
Distance to water bodies	Km	41.67	29.34	29.48	32.97	30.39	28.88	37.73	30.46
Wealth index	Score	1.62	2.194	53.63	52.79	1.606	1.259	10.93	30
Total months hh experienced food insecurity	Months per year	3.084	3.096	1.279	2.167	1.659	2.549	2.539	2.972
Household unable to reach food markets	1 unable; 0 otherwise	0.00426	0.0651	0.00239	0.0488	0.0972	0.296	0.0183	0.134
Households with cultivated and/or owned land	1 households with cultivated/ov	0.78	0.415	0.624	0.485	0.584	0.493	0.721	0.448
Employed households member	Employed over not employed	0.405	0.307	0.475	0.288	0.508	0.255	0.433	0.299
Household consumed fish in past 7 days	Household reporting to consum	0.731	0.443	0.75	0.433	0.358	0.48	0.677	0.468

Supplementary Table 3a Fish and other animal-source food consumption by proximity to water bodies

		National			Rural			Urban		
					Malawi					
**Food item**	**Measure**	**>5 Km.**	**<=5 Km**	**Total**	**>5 Km.**	**<=5 Km**	**Total**	**>5 Km.**	**<=5 Km**	**Total**
Fish	Kg. per household per week	0.80***	1.59***	0.9	0.62***	1.62***	0.77	1.34	1.47	1.35
Fish	share of HHs who consumed over 7 days (*100)	0.72***	0.89***	0.73	0.67***	0.88***	0.7	0.89	0.93	0.89
Fish	Number of days consumed (out of 7)	1.62***	2.90***	1.77	1.51***	3.00***	1.71	1.97**	2.24**	1.99
Poultry	Kg. per household per week	0.3	0.23	0.29	0.13*	0.23*	0.14	0.50**	0.24**	0.49
Poultry	share of HHs who consumed over 7 days (*100)	0.14***	0.07***	0.13	0.09***	0.05***	0.09	0.31***	0.15***	0.3
Poultry	Number of days consumed (out of 7)	0.09***	0.02***	0.08	0.05***	0.02***	0.05	0.21***	0.06***	0.2
Goat	Kg. per household per week	0.95	0.93	0.95	0.94	0.93	0.94	0.98	0.9	0.97
Goat	share of HHs who consumed over 7 days (*100)	0.14***	0.08***	0.13	0.12***	0.07***	0.11	0.22***	0.14***	0.22
Goat	Number of days consumed (out of 7)	0.28***	0.12***	0.26	0.26***	0.11***	0.24	0.34***	0.17***	0.32
Pork	Kg. per household per week	0.71**	0.96**	0.72	0.69**	0.96**	0.7	0.79	0.95	0.8
Pork	share of HHs who consumed over 7 days (*100)	0.07***	0.02***	0.07	0.07***	0.02***	0.06	0.09***	0.03***	0.08
Pork	Number of days consumed (out of 7)	0.12***	0.02***	0.11	0.12***	0.02***	0.11	0.11**	0.03**	0.1
Beef	Kg. per household per week	1.09	1.12	1.09	1.02	1.11	1.03	1.13	1.13	1.13
Beef	share of HHs who consumed over 7 days (*100)	0.10***	0.06***	0.09	0.05	0.04	0.05	0.31***	0.17***	0.3
Beef	Number of days consumed (out of 7)	0.20***	0.11***	0.19	0.1	0.08	0.1	0.52***	0.27***	0.5
Eggs	Kg. per household per week	0.40**	0.37**	0.4	0.35	0.36	0.35	0.49**	0.40**	0.49
Eggs	share of HHs who consumed over 7 days (*100)	0.32***	0.24***	0.31	0.24**	0.21**	0.23	0.65***	0.43***	0.63
Eggs	Number of days consumed (out of 7)	0.41***	0.23***	0.38	0.32***	0.20***	0.31	0.67***	0.38***	0.65
					Tanzania					
Fish	Kg. per household per week	0.73***	1.82***	0.98	0.71***	2.09***	0.96	0.76***	1.57***	1.03
Fish	share of HHs who consumed over 7 days (*100)	0.68***	0.82***	0.71	0.66***	0.82***	0.68	0.75***	0.83***	0.78
Fish	Number of days consumed (out of 7)	1.65***	3.04***	1.96	1.70***	3.46***	1.99	1.56***	2.64***	1.9
Poultry	Kg. per household per week	1.34*	1.23*	1.32	1.52	1.37	1.5	1.01	1.11	1.04
Poultry	share of HHs who consumed over 7 days (*100)	0.17	0.18	0.17	0.15	0.17	0.16	0.2	0.2	0.2
Poultry	Number of days consumed (out of 7)	0.37	0.36	0.36	0.4	0.36	0.39	0.3	0.35	0.31
Goat	Kg. per household per week	1	1.05	1.01	1.03	1.17	1.05	0.87	0.83	0.86
Goat	share of HHs who consumed over 7 days (*100)	0.13***	0.09***	0.12	0.16*	0.12*	0.15	0.08	0.06	0.07
Goat	Number of days consumed (out of 7)	0.30***	0.14***	0.27	0.37**	0.22**	0.35	0.14**	0.07**	0.12
Pork	Kg. per household per week	0.82	0.95	0.83	0.82	0.5	0.81	0.84	1.2	0.9
Pork	share of HHs who consumed over 7 days (*100)	0.05***	0.01***	0.04	0.05***	0.01***	0.04	0.04**	0.02**	0.03
Pork	Number of days consumed (out of 7)	0.08***	0.02***	0.07	0.10***	0.01***	0.08	0.05	0.02	0.04
Beef	Kg. per household per week	1.04	1.07	1.04	1.05	1.04	1.05	1.03	1.08	1.04
Beef	share of HHs who consumed over 7 days (*100)	0.39**	0.44**	0.4	0.28	0.28	0.28	0.64**	0.59**	0.63
Beef	Number of days consumed (out of 7)	0.88	0.87	0.87	0.65***	0.46***	0.62	1.37	1.26	1.34
Eggs	Kg. per household per week	0.39	0.44	0.4	0.33	0.38	0.34	0.45	0.47	0.46
Eggs	share of HHs who consumed over 7 days (*100)	0.2	0.2	0.2	0.15	0.14	0.15	0.31*	0.26*	0.29
Eggs	Number of days consumed (out of 7)	0.18	0.16	0.18	0.14	0.1	0.13	0.27	0.22	0.26

		National			Rural			Urban		
					Uganda					
**Food item**	**Measure**	**>5 Km.**	**<=5 Km**	**Total**	**>5 Km.**	**<=5 Km**	**Total**	**>5 Km.**	**<=5 Km**	**Total**
Fish	Kg. per household per week	1.47	1.49	1.47	1.44	1.53	1.46	1.52	1.36	1.5
Fish	share of HHs who consumed over 7 days (*100)	0.31***	0.46***	0.33	0.29***	0.47***	0.31	0.39	0.43	0.4
Fish	Number of days consumed (out of 7)	1.11***	2.31***	1.3	1.15***	2.67***	1.4	1.04**	1.36**	1.09
Poultry	Kg. per household per week	1.67**	1.14**	1.56	1.94**	1.12**	1.74	1.51	1.16	1.45
Poultry	share of HHs who consumed over 7 days (*100)	0.02	0.04	0.03	0.01	0.02	0.01	0.06	0.09	0.07
Poultry	Number of days consumed (out of 7)	0.18	0.17	0.18	0.15	0.17	0.16	0.22	0.18	0.22
Goat	Kg. per household per week	1.09	0.91	1.06	1.09*	0.76*	1.03	1.07**	2.07**	1.16
Goat	share of HHs who consumed over 7 days (*100)	0.04	0.05	0.04	0.04	0.06	0.05	0.04	0.02	0.04
Goat	Number of days consumed (out of 7)	0.12	0.13	0.12	0.14	0.15	0.14	0.09	0.06	0.08
Pork	Kg. per household per week	1.08	0.95	1.06	1.1	0.95	1.08	0.95	1.01	0.96
Pork	share of HHs who consumed over 7 days (*100)	0.06**	0.03**	0.06	0.07*	0.04*	0.06	0.04	0.01	0.03
Pork	Number of days consumed (out of 7)	0.18***	0.07***	0.16	0.21***	0.09***	0.19	0.1	0.03	0.09
Beef	Kg. per household per week	1.42	1.39	1.42	1.3	1.36	1.31	1.65	1.44	1.63
Beef	share of HHs who consumed over 7 days (*100)	0.32***	0.21***	0.3	0.27***	0.18***	0.26	0.46***	0.28***	0.43
Beef	Number of days consumed (out of 7)	1.07***	0.60***	0.99	0.94***	0.53***	0.87	1.34***	0.78***	1.26
Eggs	Kg. per household per week	0.42	0.45	0.43	0.35	0.36	0.35	0.49	0.52	0.5
Eggs	share of HHs who consumed over 7 days (*100)	0.09	0.09	0.09	0.06	0.05	0.06	0.19	0.2	0.19
Eggs	Number of days consumed (out of 7)	0.59	0.53	0.58	0.4	0.27	0.38	1	1.22	1.03
					All countries					
Fish	Kg. per household per week	0.86***	1.74***	1.04	0.79***	1.88***	0.98	1.00***	1.54***	1.15
Fish	share of HHs who consumed over 7 days (*100)	0.59***	0.75***	0.61	0.56***	0.73***	0.58	0.68***	0.77***	0.7
Fish	Number of days consumed (out of 7)	1.54***	2.90***	1.79	1.54***	3.20***	1.8	1.52***	2.49***	1.76
Poultry	Kg. per household per week	1.11	1.16	1.12	1.23	1.24	1.24	0.91***	1.09***	0.96
Poultry	share of HHs who consumed over 7 days (*100)	0.12**	0.14**	0.12	0.1	0.11	0.1	0.18	0.18	0.18
Poultry	Number of days consumed (out of 7)	0.26	0.28	0.27	0.26	0.25	0.26	0.27**	0.32**	0.28
Goat	Kg. per household per week	1	1.02	1	1.02	1.06	1.02	0.93	0.91	0.93
Goat	share of HHs who consumed over 7 days (*100)	0.11***	0.08***	0.1	0.12***	0.09***	0.11	0.09***	0.06***	0.08
Goat	Number of days consumed (out of 7)	0.26***	0.14***	0.24	0.30***	0.18***	0.28	0.17***	0.08***	0.14
Pork	Kg. per household per week	0.87	0.95	0.87	0.87	0.85	0.87	0.85**	1.15**	0.88
Pork	share of HHs who consumed over 7 days (*100)	0.06***	0.02***	0.05	0.06***	0.02***	0.05	0.04***	0.02***	0.04
Pork	Number of days consumed (out of 7)	0.11***	0.03***	0.09	0.13***	0.03***	0.11	0.07***	0.02***	0.06
Beef	Kg. per household per week	1.15	1.11	1.15	1.13	1.13	1.13	1.18	1.11	1.16
Beef	share of HHs who consumed over 7 days (*100)	0.31***	0.34***	0.31	0.23**	0.21**	0.22	0.54	0.52	0.54
Beef	Number of days consumed (out of 7)	0.76*	0.72*	0.75	0.57***	0.39***	0.55	1.21	1.16	1.2
Eggs	Kg. per household per week	0.40**	0.43**	0.4	0.34***	0.37***	0.34	0.47	0.47	0.47
Eggs	share of HHs who consumed over 7 days (*100)	0.19*	0.18*	0.19	0.15**	0.13**	0.14	0.33***	0.26***	0.31
Eggs	Number of days consumed (out of 7)	0.31***	0.23***	0.3	0.23***	0.16***	0.22	0.50***	0.34***	0.46

* significant at 10%; ** significant at 5%; *** significant at 1%

Supplementary Table 3b Fish and other animal-source food consumption by proximity to water bodies - by poor/non-poor HHs (above and below national poverty lines)

		National			<=5Km			>=5Km		
		Malawi								
**Food item**	**Measure**	**Non-poor**	**Poor**	**Total**	**Non-poor**	**Poor**	**Total**	**Non-poor**	**Poor**	**Total**
Fish	Kg. per household per week	1.08***	0.62***	0.9	1.83***	1.31***	1.59	0.98***	0.50***	0.8
Fish	share of HHs who consumed over 7 days (*100)	0.82***	0.63***	0.73	0.93***	0.85***	0.89	0.81***	0.60***	0.72
Fish	Number of days consumed (out of 7)	1.79*	1.74*	1.77	2.93	2.85	2.9	1.66***	1.56***	1.62
Poultry	Kg. per household per week	0.31***	0.15***	0.29	0.22	0.3	0.23	0.31***	0.14***	0.3
Poultry	share of HHs who consumed over 7 days (*100)	0.21***	0.03***	0.13	0.11***	0.02***	0.07	0.22***	0.03***	0.14
Poultry	Number of days consumed (out of 7)	0.11***	0.02***	0.08	0.03***	0.01***	0.02	0.12***	0.03***	0.09
Goat	Kg. per household per week	0.96**	0.88**	0.95	0.93	0.93	0.93	0.97**	0.88**	0.95
Goat	share of HHs who consumed over 7 days (*100)	0.21***	0.04***	0.13	0.12***	0.03***	0.08	0.22***	0.05***	0.14
Goat	Number of days consumed (out of 7)	0.36***	0.10***	0.26	0.18***	0.05***	0.12	0.38***	0.11***	0.28
Pork	Kg. per household per week	0.74**	0.63**	0.72	0.79**	1.31**	0.96	0.74***	0.60***	0.71
Pork	share of HHs who consumed over 7 days (*100)	0.09***	0.03***	0.07	0.02	0.01	0.02	0.10***	0.03***	0.07
Pork	Number of days consumed (out of 7)	0.14***	0.06***	0.11	0.02	0.02	0.02	0.15***	0.07***	0.12
Beef	Kg. per household per week	1.10***	0.92***	1.09	1.12	1.05	1.12	1.10***	0.91***	1.09
Beef	share of HHs who consumed over 7 days (*100)	0.16***	0.02***	0.09	0.10***	0.01***	0.06	0.17***	0.02***	0.1
Beef	Number of days consumed (out of 7)	0.28***	0.04***	0.19	0.16***	0.04***	0.11	0.30***	0.04***	0.2
Eggs	Kg. per household per week	0.42***	0.31***	0.4	0.38	0.34	0.37	0.42***	0.31***	0.4
Eggs	share of HHs who consumed over 7 days (*100)	0.47***	0.12***	0.31	0.36***	0.11***	0.24	0.48***	0.12***	0.32
Eggs	Number of days consumed (out of 7)	0.50***	0.20***	0.38	0.31***	0.12***	0.23	0.52***	0.21***	0.41
		Tanzania								
Fish	Kg. per household per week	1.06***	0.88***	0.98	1.77	1.91	1.82	0.81***	0.61***	0.73
Fish	share of HHs who consumed over 7 days (*100)	0.76***	0.65***	0.71	0.84**	0.79**	0.82	0.74***	0.62***	0.68
Fish	Number of days consumed (out of 7)	1.79***	2.20***	1.96	2.78***	3.50***	3.04	1.49***	1.88***	1.65
Poultry	Kg. per household per week	1.26***	1.50***	1.32	1.16***	1.48***	1.23	1.29***	1.50***	1.34
Poultry	share of HHs who consumed over 7 days (*100)	0.22***	0.10***	0.17	0.23***	0.11***	0.18	0.22***	0.10***	0.17
Poultry	Number of days consumed (out of 7)	0.42***	0.28***	0.36	0.42***	0.25***	0.36	0.42***	0.29***	0.37
Goat	Kg. per household per week	1.06**	0.91**	1.01	1.06	1.03	1.05	1.06**	0.90**	1
Goat	share of HHs who consumed over 7 days (*100)	0.15***	0.10***	0.12	0.10*	0.07*	0.09	0.16***	0.10***	0.13
Goat	Number of days consumed (out of 7)	0.30***	0.21***	0.27	0.16	0.12	0.14	0.35***	0.24***	0.3
Pork	Kg. per household per week	0.86	0.76	0.83	1.01	0.5	0.95	0.84	0.77	0.82
Pork	share of HHs who consumed over 7 days (*100)	0.05***	0.02***	0.04	0.02*	0.00*	0.01	0.06***	0.03***	0.05
Pork	Number of days consumed (out of 7)	0.08*	0.05*	0.07	0.02	0.01	0.02	0.09	0.06	0.08
Beef	Kg. per household per week	1.06	0.99	1.05	1.08	1	1.07	1.05	0.99	1.04
Beef	share of HHs who consumed over 7 days (*100)	0.56***	0.19***	0.4	0.57***	0.21***	0.44	0.56***	0.19***	0.39
Beef	Number of days consumed (out of 7)	1.15***	0.47***	0.88	1.14***	0.39***	0.87	1.15***	0.49***	0.88
Eggs	Kg. per household per week	0.43***	0.26***	0.4	0.46*	0.30*	0.44	0.42***	0.25***	0.38
Eggs	share of HHs who consumed over 7 days (*100)	0.29***	0.08***	0.2	0.28***	0.06***	0.2	0.29***	0.09***	0.2
Eggs	Number of days consumed (out of 7)	0.24***	0.08***	0.18	0.23***	0.05***	0.16	0.24***	0.08***	0.18

					Uganda					
Fish	Kg. per household per week	1.57***	0.93***	1.47	1.64***	0.77***	1.49	1.55***	0.98***	1.47
Fish	share of HHs who consumed over 7 days (*100)	0.36***	0.24***	0.34	0.49**	0.37**	0.46	0.34***	0.21***	0.31
Fish	Number of days consumed (out of 7)	1.28	1.44	1.3	2.32	2.26	2.31	1.1	1.24	1.12
Poultry	Kg. per household per week	1.62*	0.89*	1.58	1.28	0.46	1.11	1.7	1.76	1.7
Poultry	share of HHs who consumed over 7 days (*100)	0.03***	0.01***	0.03	0.04	0.04	0.04	0.03***	0.00***	0.02
Poultry	Number of days consumed (out of 7)	0.19***	0.07***	0.17	0.19	0.08	0.17	0.19***	0.06***	0.17
Goat	Kg. per household per week	1.10**	0.57**	1.06	0.93	0.44	0.91	1.14**	0.58**	1.09
Goat	share of HHs who consumed over 7 days (*100)	0.05***	0.02***	0.04	0.06*	0.01*	0.05	0.05***	0.02***	0.04
Goat	Number of days consumed (out of 7)	0.13**	0.06**	0.12	0.15*	0.02*	0.13	0.13	0.06	0.12
Pork	Kg. per household per week	1.10**	0.72**	1.06	0.94	1.09	0.95	1.12**	0.70**	1.07
Pork	share of HHs who consumed over 7 days (*100)	0.06***	0.03***	0.06	0.04	0.01	0.03	0.07***	0.04***	0.06
Pork	Number of days consumed (out of 7)	0.17	0.1	0.16	0.07	0.06	0.07	0.18	0.11	0.18
Beef	Kg. per household per week	1.48***	0.80***	1.42	1.46*	0.89*	1.39	1.48***	0.78***	1.43
Beef	share of HHs who consumed over 7 days (*100)	0.35***	0.12***	0.3	0.23**	0.12**	0.21	0.37***	0.12***	0.32
Beef	Number of days consumed (out of 7)	1.07***	0.46***	0.99	0.65*	0.36*	0.6	1.14***	0.49***	1.07
Eggs	Kg. per household per week	0.43	0.22	0.43	0.46	0.05	0.45	0.43	0.23	0.42
Eggs	share of HHs who consumed over 7 days (*100)	0.11***	0.01***	0.09	0.11***	0.01***	0.09	0.12***	0.01***	0.09
Eggs	Number of days consumed (out of 7)	0.65***	0.08***	0.58	0.63**	0.01**	0.54	0.66***	0.09***	0.59
					All countries					
Fish	Kg. per household per week	1.16***	0.82***	1.04	1.75	1.71	1.74	1.00***	0.60***	0.86
Fish	share of HHs who consumed over 7 days (*100)	0.63***	0.59***	0.62	0.75	0.74	0.75	0.61***	0.56***	0.59
Fish	Number of days consumed (out of 7)	1.66***	2.04***	1.79	2.70***	3.29***	2.9	1.42***	1.76***	1.54
Poultry	Kg. per household per week	1.05***	1.36***	1.12	1.10***	1.37***	1.16	1.04***	1.36***	1.11
Poultry	share of HHs who consumed over 7 days (*100)	0.16***	0.07***	0.12	0.16***	0.08***	0.14	0.15***	0.07***	0.12
Poultry	Number of days consumed (out of 7)	0.30***	0.20***	0.27	0.33***	0.19***	0.28	0.29***	0.21***	0.26
Goat	Kg. per household per week	1.04***	0.90***	1	1.02	1.01	1.02	1.04***	0.88***	1
Goat	share of HHs who consumed over 7 days (*100)	0.13***	0.07***	0.1	0.09***	0.05***	0.08	0.13***	0.08***	0.11
Goat	Number of days consumed (out of 7)	0.27***	0.18***	0.24	0.16***	0.10***	0.14	0.30***	0.19***	0.26
Pork	Kg. per household per week	0.91***	0.72***	0.87	0.95	0.96	0.95	0.91***	0.71***	0.86
Pork	share of HHs who consumed over 7 days (*100)	0.06***	0.03***	0.05	0.02***	0.01***	0.02	0.07***	0.03***	0.06
Pork	Number of days consumed (out of 7)	0.11***	0.06***	0.09	0.03**	0.01**	0.03	0.13***	0.07***	0.11
Beef	Kg. per household per week	1.18***	0.96***	1.15	1.14*	0.99*	1.11	1.19***	0.96***	1.15
Beef	share of HHs who consumed over 7 days (*100)	0.42***	0.14***	0.31	0.43***	0.16***	0.34	0.42***	0.14***	0.31
Beef	Number of days consumed (out of 7)	0.95***	0.37***	0.75	0.92***	0.32***	0.72	0.96***	0.38***	0.76
Eggs	Kg. per household per week	0.43***	0.27***	0.4	0.44***	0.31***	0.43	0.42***	0.27***	0.4
Eggs	share of HHs who consumed over 7 days (*100)	0.26***	0.08***	0.19	0.24***	0.06***	0.18	0.26***	0.08***	0.19
Eggs	Number of days consumed (out of 7)	0.40***	0.11***	0.3	0.32***	0.06***	0.23	0.42***	0.12***	0.31

* significant at 10%; ** significant at 5%; *** significant at 1%

Kg. per household per week : average based on households who reported to consume any food item

Number of days consumed (out of 7): average based on households who reported to consume at least some Qt. of any food from animal source

Supplementary Table 3c Fish and other animal-source food consumption by proximity to water bodies - by dietary diversity score (food consumption score)

		National				<=5Km.				>5Km.			
		Malawi											
**Food item**	**Measure**	**Poor**	**Borderline**	**Acceptable**	**Total**	**Poor**	**Borderline**	**Acceptable**	**Total**	**Poor**	**Borderline**	**Acceptable**	**Total**
**Fish**	**Kg. per household per week**	**0.40*** **	**0.56*** **	**1.22*** **	**0.9**	**0.86*** **	**1.09*** **	**2.00*** **	**1.59**	**0.36*** **	**0.48*** **	**1.10*** **	**0.8**
Fish	share of HHs who consumed over 7 days (*10	0.40***	0.71***	0.87***	0.73	0.45***	0.89	0.98***	0.89	0.40***	0.69***	0.86***	0.72
Fish	Number of days consumed (out of 7)	0.73***	1.37***	2.23***	1.77	0.78***	2.21***	3.57***	2.9	0.73***	1.26***	2.04***	1.62
Poultry	Kg. per household per week	0.16	0.14***	0.33***	0.29	.	0.31	0.21	0.23	0.16	0.12***	0.34***	0.3
Poultry	share of HHs who consumed over 7 days (*10	0.02***	0.05***	0.23***	0.13	0.00***	0.04***	0.10***	0.07	0.02***	0.06***	0.25***	0.14
Poultry	Number of days consumed (out of 7)	0.02***	0.03***	0.13***	0.08	0	0.01	0.03*	0.02	0.02***	0.03***	0.14***	0.09
Goat	Kg. per household per week	0.78**	0.90**	0.97***	0.95	1	0.84	0.94	0.93	0.77**	0.90**	0.97***	0.95
Goat	share of HHs who consumed over 7 days (*10	0.02***	0.07***	0.23***	0.13	0.02***	0.03***	0.12***	0.08	0.02***	0.08***	0.24***	0.14
Goat	Number of days consumed (out of 7)	0.04***	0.12***	0.40***	0.26	0.03*	0.05***	0.18***	0.12	0.04***	0.13***	0.44***	0.28
Pork	Kg. per household per week	0.50***	0.63***	0.78***	0.72	.	0.97	0.96	0.96	0.50**	0.62***	0.77***	0.71
Pork	share of HHs who consumed over 7 days (*10	0.02***	0.05***	0.09***	0.07	0.00*	0.02	0.03*	0.02	0.02***	0.06***	0.10***	0.07
Pork	Number of days consumed (out of 7)	0.03***	0.08***	0.14***	0.11	0	0.02	0.03	0.02	0.04***	0.09***	0.16***	0.12
Beef	Kg. per household per week	0.77	0.93***	1.12***	1.09	1	0.86***	1.17***	1.12	0.68	0.94***	1.11***	1.09
Beef	share of HHs who consumed over 7 days (*10	0.00***	0.03***	0.18***	0.09	0.01***	0.03***	0.10***	0.06	0.00***	0.03***	0.19***	0.1
Beef	Number of days consumed (out of 7)	0.01***	0.05***	0.32***	0.19	0.02*	0.05***	0.15***	0.11	0.00***	0.05***	0.35***	0.2
Eggs	Kg. per household per week	0.25***	0.30***	0.44***	0.4	0.35	0.27***	0.40***	0.37	0.24***	0.30***	0.44***	0.4
Eggs	share of HHs who consumed over 7 days (*10	0.05***	0.18***	0.51***	0.31	0.05***	0.13***	0.36***	0.24	0.05***	0.19***	0.53***	0.32
Eggs	Number of days consumed (out of 7)	0.08***	0.19***	0.57***	0.38	0.10**	0.10***	0.32***	0.23	0.08***	0.20***	0.61***	0.41
		Tanzania											
**Fish**	**Kg. per household per week**	**0.33*** **	**0.41*** **	**1.08*** **	**0.98**	**0.48** **	**0.69*** **	**1.93*** **	**1.82**	**0.29*** **	**0.37*** **	**0.80*** **	**0.73**
Fish	share of HHs who consumed over 7 days (*10	0.23***	0.56***	0.77***	0.71	0.43***	0.64***	0.86***	0.82	0.20***	0.54***	0.75***	0.68
Fish	Number of days consumed (out of 7)	0.82***	1.20***	2.10***	1.96	0.79***	1.46***	3.22***	3.04	0.82***	1.15***	1.76***	1.65
Poultry	Kg. per household per week	1.51	1.18	1.33	1.32	0.5	0.94*	1.25*	1.23	1.53	1.22	1.36	1.34
Poultry	share of HHs who consumed over 7 days (*10	0.03***	0.09***	0.19***	0.17	0.00**	0.12*	0.20***	0.18	0.03***	0.09***	0.19***	0.17
Poultry	Number of days consumed (out of 7)	0.06**	0.10***	0.41***	0.36	0.02	0.13**	0.38***	0.36	0.06*	0.09***	0.42***	0.37
Goat	Kg. per household per week	0.35	0.64***	1.05***	1.01	.	0.58	1.07	1.05	0.35	0.65***	1.05***	1
Goat	share of HHs who consumed over 7 days (*10	0.00***	0.08***	0.14***	0.12	0	0.03**	0.10***	0.09	0.00***	0.08***	0.15***	0.13
Goat	Number of days consumed (out of 7)	0.00**	0.13***	0.29***	0.27	0	0.03*	0.16**	0.14	0.00**	0.14***	0.34***	0.3
Pork	Kg. per household per week	0.71	1.08	0.82	0.83	.	.	0.95	0.95	0.71	1.08	0.81	0.82
Pork	share of HHs who consumed over 7 days (*10	0.02	0.01***	0.05***	0.04	0	0	0.01	0.01	0.02	0.01***	0.06***	0.05
Pork	Number of days consumed (out of 7)	0	0.01***	0.08***	0.07	0	0	0.02	0.02	0	0.01***	0.10***	0.08
Beef	Kg. per household per week	0.52	0.67***	1.08***	1.04	1	0.62***	1.10***	1.07	0.5	0.68***	1.07***	1.04
Beef	share of HHs who consumed over 7 days (*10	0.02***	0.20***	0.46***	0.4	0.01***	0.28***	0.47***	0.44	0.03***	0.19***	0.46***	0.39
Beef	Number of days consumed (out of 7)	0.07***	0.25***	1.00***	0.87	0.02**	0.27***	0.94***	0.87	0.08***	0.25***	1.01***	0.88
Eggs	Kg. per household per week	0.19	0.25***	0.41***	0.4	0.19	0.28	0.45	0.44	0.19	0.25***	0.40***	0.39
Eggs	share of HHs who consumed over 7 days (*10	0.01***	0.08***	0.23***	0.2	0.04**	0.11**	0.22***	0.2	0.00***	0.08***	0.24***	0.2
Eggs	Number of days consumed (out of 7)	0.01**	0.06***	0.20***	0.18	0.05	0.05**	0.18***	0.16	0.00**	0.06***	0.21***	0.18

		Uganda											
**Fish**	**Kg. per household per week**	**0.44**	**0.75*** **	**1.52*** **	**1.47**	**0.44**	**0.37** **	**1.55** **	**1.49**	**.**	**0.83*** **	**1.51*** **	**1.47**
Fish	share of HHs who consumed over 7 days (*10	0.00***	0.15***	0.39***	0.34	0.02***	0.24***	0.52***	0.46	0.00***	0.13***	0.37***	0.31
Fish	Number of days consumed (out of 7)	0	0.73***	1.34***	1.3	0	1.12**	2.39**	2.31	0	0.67***	1.15***	1.12
Poultry	Kg. per household per week	.	.	1.58	1.58	.	.	1.11	1.11	.	.	1.7	1.7
Poultry	share of HHs who consumed over 7 days (*10	0.00**	0.00***	0.03***	0.03	0	0	0.05*	0.04	0.00**	0.00***	0.03***	0.02
Poultry	Number of days consumed (out of 7)	0	0.00***	0.19***	0.17	0	0	0.18	0.17	0	0.00***	0.19***	0.17
Goat	Kg. per household per week	.	0.82	1.06	1.06	.	.	0.91	0.91	.	0.82	1.09	1.09
Goat	share of HHs who consumed over 7 days (*10	0.00***	0.01***	0.05***	0.04	0	0	0.06**	0.05	0.00***	0.01***	0.05***	0.04
Goat	Number of days consumed (out of 7)	0	0.03**	0.13**	0.12	0	0	0.14	0.13	0	0.03*	0.13*	0.12
Pork	Kg. per household per week	.	0.69*	1.08*	1.06	.	.	0.95	0.95	.	0.69*	1.09*	1.07
Pork	share of HHs who consumed over 7 days (*10	0.00***	0.02***	0.07***	0.06	0	0	0.04*	0.03	0.00***	0.03***	0.07***	0.06
Pork	Number of days consumed (out of 7)	0	0.08	0.17	0.16	0	0	0.08	0.07	0	0.09	0.18	0.18
Beef	Kg. per household per week	.	0.82***	1.45***	1.42	.	0.82	1.41	1.39	.	0.82***	1.45***	1.43
Beef	share of HHs who consumed over 7 days (*10	0.00***	0.10***	0.36***	0.31	0.00***	0.10*	0.24***	0.21	0.00***	0.10***	0.38***	0.32
Beef	Number of days consumed (out of 7)	0	0.45***	1.03***	0.99	0	0.28	0.62	0.6	0	0.48***	1.11***	1.07
Eggs	Kg. per household per week	0.08	0.35	0.43	0.43	.	0.82*	0.42*	0.45	0.08	0.23*	0.44**	0.42
Eggs	share of HHs who consumed over 7 days (*10	0.01***	0.04***	0.11***	0.09	0.00*	0.07	0.1	0.09	0.02***	0.03***	0.11***	0.09
Eggs	Number of days consumed (out of 7)	0.8	0.31**	0.60**	0.58	0	0.62	0.53	0.54	1	0.26**	0.61**	0.59
		All countries											
**Fish**	**Kg. per household per week**	**0.38*** **	**0.51*** **	**1.18*** **	**1.04**	**0.65*** **	**0.87*** **	**1.88*** **	**1.74**	**0.34*** **	**0.45*** **	**0.98*** **	**0.86**
Fish	share of HHs who consumed over 7 days (*10	0.25***	0.55***	0.67***	0.62	0.29***	0.66***	0.79***	0.75	0.25***	0.53***	0.64***	0.59
Fish	Number of days consumed (out of 7)	0.75***	1.25***	1.95***	1.79	0.77***	1.76***	3.11***	2.9	0.75***	1.17***	1.66***	1.54
Poultry	Kg. per household per week	0.98	0.82***	1.16***	1.12	0.5	0.82**	1.18**	1.16	0.98	0.82***	1.15***	1.11
Poultry	share of HHs who consumed over 7 days (*10	0.02***	0.06***	0.15***	0.12	0.00***	0.07***	0.15***	0.14	0.02***	0.06***	0.15***	0.12
Poultry	Number of days consumed (out of 7)	0.03***	0.06***	0.32***	0.27	0.01**	0.07***	0.32***	0.28	0.03***	0.06***	0.32***	0.26
Goat	Kg. per household per week	0.72*	0.76***	1.04***	1	1	0.69*	1.03	1.02	0.70*	0.77***	1.04***	1
Goat	share of HHs who consumed over 7 days (*10	0.01***	0.06***	0.13***	0.11	0.01***	0.03***	0.09***	0.08	0.01***	0.07***	0.13***	0.11
Goat	Number of days consumed (out of 7)	0.03***	0.11***	0.27***	0.24	0.01	0.04***	0.16***	0.14	0.03***	0.13***	0.30***	0.26
Pork	Kg. per household per week	0.59**	0.70***	0.90***	0.87	.	0.97	0.95	0.95	0.59**	0.70***	0.89***	0.86
Pork	share of HHs who consumed over 7 days (*10	0.01***	0.03***	0.06***	0.05	0	0.01*	0.02**	0.02	0.02***	0.03***	0.07***	0.06
Pork	Number of days consumed (out of 7)	0.02***	0.04***	0.11***	0.09	0	0.01	0.03*	0.03	0.02***	0.05***	0.13***	0.11
Beef	Kg. per household per week	0.55*	0.72***	1.18***	1.15	1	0.65***	1.14***	1.11	0.52*	0.73***	1.19***	1.15
Beef	share of HHs who consumed over 7 days (*10	0.01***	0.12***	0.40***	0.31	0.01***	0.16***	0.38***	0.34	0.01***	0.11***	0.40***	0.31
Beef	Number of days consumed (out of 7)	0.03***	0.17***	0.91***	0.75	0.02***	0.18***	0.81***	0.72	0.03***	0.17***	0.93***	0.76
Eggs	Kg. per household per week	0.22***	0.29***	0.42***	0.4	0.27	0.33*	0.44**	0.43	0.22***	0.28***	0.42***	0.4
Eggs	share of HHs who consumed over 7 days (*10	0.03***	0.11***	0.23***	0.19	0.03***	0.11***	0.20***	0.18	0.03***	0.12***	0.24***	0.19
Eggs	Number of days consumed (out of 7)	0.06***	0.14***	0.34***	0.3	0.07	0.11***	0.25***	0.23	0.06***	0.14***	0.37***	0.31

* significant at 10%; ** significant at 5%; *** significant at 1%

Supplementary Table 4a Fish consumption - form (dried/fresh) and source (purchased/own production/other) - by proximity to water bodies

			**National**			**Rural**			**Urban**		
						Malawi					
**Fish consumption by:**	**Fish consumption**	**Indicator**	**>5 Km.**	**<=5 Km**	**Total**	**>5 Km.**	**<=5 Km**	**Total**	**>5 Km.**	**<=5 Km**	**Total**
Form	Total fish	% of total household	0.72***	0.89***	0.73	0.67***	0.88***	0.7	0.89	0.93	0.89
	Fresh fish	% of total household who consumed fish	0.22***	0.73***	0.28	0.16***	0.74***	0.25	0.38***	0.70***	0.41
	Dried fish	% of total household who consumed fish	0.74***	0.49***	0.71	0.73***	0.49***	0.7	0.77***	0.52***	0.75
	Other fish	% of total household who consumed fish	0.30***	0.20***	0.28	0.26***	0.19***	0.25	0.40***	0.27***	0.38
	Total fish	Kg. per household /per week	0.80***	1.59***	0.9	0.62***	1.62***	0.77	1.34	1.47	1.35
	Fresh fish	Kg. per household /per week ****	1.36**	1.53**	1.42	1.13***	1.56***	1.32	1.67**	1.29**	1.62
	Dried fish	Kg. per household /per week ****	0.49***	0.73***	0.51	0.43***	0.73***	0.46	0.66	0.7	0.66
	Other fish	Kg. per household /per week ****	0.48***	0.60***	0.49	0.46**	0.57**	0.47	0.51**	0.74**	0.53
Source	From purchased	Kg. per household /per week ****	0.79***	1.29***	0.85	0.61***	1.28***	0.7	1.34	1.36	1.34
	From own production	Kg. per household /per week ****	1.95***	3.27***	2.77	1.97***	3.38***	2.84	1.62	1.83	1.77
	From other source (including gift)	Kg. per household /per week ****	0.61***	2.26***	1.23	0.60***	2.23***	1.26	0.66***	2.50***	1.03
	From purchased	% of total fish consumed	0.97***	0.86***	0.96	0.97***	0.85***	0.95	0.98***	0.92***	0.98
	From own production	% of total fish consumed	0.00***	0.05***	0.01	0.01***	0.06***	0.01	0.00***	0.03***	0
	From other source (including gift)	% of total fish consumed	0.02***	0.09***	0.03	0.03***	0.09***	0.04	0.02***	0.06***	0.02
						Tanzania					
Form	Total fish	% of total household	0.68***	0.82***	0.71	0.66***	0.82***	0.68	0.75***	0.83***	0.78
	Fresh fish	% of total household who consumed fish	0.67***	0.87***	0.71	0.64***	0.87***	0.68	0.72***	0.87***	0.77
	Dried fish	% of total household who consumed fish	0.49***	0.36***	0.46	0.52***	0.40***	0.5	0.43***	0.32***	0.4
	Other fish	% of total household who consumed fish	0.00*	0.00*	0						
	Total fish	Kg. per household /per week	0.73***	1.82***	0.98	0.71***	2.09***	0.96	0.76***	1.57***	1.03
	Fresh fish	Kg. per household /per week ****	0.71***	1.77***	1.01	0.68***	2.01***	0.98	0.78***	1.55***	1.07
	Dried fish	Kg. per household /per week ****	0.52***	0.76***	0.56	0.54***	0.83***	0.59	0.46***	0.67***	0.51
	Other fish	Kg. per household /per week ****	.	0.81	0.81						
Source	From purchased	Kg. per household /per week ****	0.70***	1.60***	0.9	0.68***	1.87***	0.88	0.75***	1.35***	0.94
	From own production	Kg. per household /per week ****	2.56**	4.16**	3.5	2.79	3.39	3.09	0.50**	5.51**	4.7
	From other source (including gift)	Kg. per household /per week ****	1.11***	2.56***	1.86	1.10***	2.54***	1.76	1.15*	2.59*	2.08
	From purchased	% of total fish consumed	0.97***	0.89***	0.95	0.97***	0.86***	0.95	0.98***	0.92***	0.96
	From own production	% of total fish consumed	0.01***	0.03***	0.01	0.01***	0.04***	0.01	0.00***	0.02***	0.01
	From other source (including gift)	% of total fish consumed	0.02***	0.08***	0.04	0.03***	0.10***	0.04	0.02***	0.06***	0.03
						Uganda					
Form	Total fish	% of total household	0.31***	0.46***	0.33	0.29***	0.47***	0.31	0.39	0.43	0.4
	Fresh fish	% of total household who consumed fish	0.44***	0.76***	0.51	0.39***	0.83***	0.49	0.56	0.53	0.55
	Dried fish	% of total household who consumed fish	0.70***	0.42***	0.64	0.71***	0.39***	0.64	0.66*	0.52*	0.64
	Other fish	% of total household who consumed fish	N.A.	N.A.	N.A.	N.A.	N.A.	N.A.	N.A.	N.A.	N.A.
	Total fish	Kg. per household /per week	1.47	1.49	1.47	1.44	1.53	1.46	1.52	1.36	1.5
	Fresh fish	Kg. per household /per week ****	0.83**	1.01**	0.88	0.83**	1.02**	0.9	0.83	0.96	0.85
	Dried fish	Kg. per household /per week ****	1.58	1.71	1.6	1.57	1.73	1.59	1.61	1.64	1.61

	Other fish	Kg. per household /per week ****	N.A.	N.A.	N.A.	N.A.	N.A.	N.A.	N.A.	N.A.	N.A.
Source	From purchased	Kg. per household /per week ****	1.46	1.37	1.45	1.42	1.38	1.41	1.55	1.37	1.52
	From own production	Kg. per household /per week ****	1.24	1.77	1.59	1.08	1.77	1.54	3.54		3.54
	From other source (including gift)	Kg. per household /per week ****	1.43	1.61	1.49	1.65	1.65	1.65	0.88	0.71	0.87
	From purchased	% of total fish consumed	0.94***	0.84***	0.92	0.93***	0.79***	0.9	0.95	0.99	0.96
	From own production	% of total fish consumed	0.01***	0.09***	0.03	0.02***	0.11***	0.04	0	0	0
	From other source (including gift)	% of total fish consumed	0.05*	0.08*	0.05	0.05**	0.10**	0.06	0.05	0.01	0.04
						All countries					
Form	Total fish	% of total household	0.59***	0.75***	0.61	0.56***	0.73***	0.58	0.68***	0.77***	0.7
	Fresh fish	% of total household who consumed fish	0.52***	0.83***	0.58	0.47***	0.83***	0.53	0.63***	0.84***	0.68
	Dried fish	% of total household who consumed fish	0.59***	0.39***	0.55	0.61***	0.42***	0.57	0.54***	0.35***	0.49
	Other fish	% of total household who consumed fish	0.08***	0.03***	0.07	0.07***	0.04***	0.07	0.08***	0.02***	0.07
	Total fish	Kg. per household /per week	0.86***	1.74***	1.04	0.79***	1.88***	0.98	1.00***	1.54***	1.15
	Fresh fish	Kg. per household /per week ****	0.80***	1.64***	1.04	0.74***	1.74***	1.01	0.90***	1.50***	1.1
	Dried fish	Kg. per household /per week ****	0.69***	0.90***	0.72	0.68***	0.97***	0.72	0.73	0.79	0.74
	Other fish	Kg. per household /per week ****	0.48***	0.61***	0.49	0.46***	0.58***	0.48	0.51**	0.71**	0.52
Source	From purchased	Kg. per household /per week ****	0.83***	1.52***	0.97	0.76***	1.65***	0.91	1.00***	1.35***	1.09
	From own production	Kg. per household /per week ****	2.09***	3.24***	2.8	2.17**	2.76**	2.51	1.30***	5.24***	4.39
	From other source (including gift)	Kg. per household /per week ****	1.07***	2.37***	1.65	1.10***	2.30***	1.61	0.97***	2.55***	1.76
	From purchased	% of total fish consumed	0.97***	0.88***	0.95	0.96***	0.85***	0.94	0.98***	0.93***	0.96
	From own production	% of total fish consumed	0.01***	0.04***	0.01	0.01***	0.06***	0.02	0.00***	0.02***	0.01
	From other source (including gift)	% of total fish consumed	0.03***	0.08***	0.04	0.03***	0.10***	0.04	0.02***	0.05***	0.03

* significant at 10%; ** significant at 5%; *** significant at 1%

Kg. per household per week : average based on households who reported to consume fish

**** averaged among households who reported to consume a given form or source of fish. 0 are not included in the calculation

value is missing if insufficient observations for statistically significant average

Supplementary Table 4b Fish consumption - form (dried/fresh) and source (purchased/own production/other) - by proximity to water bodies and poor/non-poor HHs (above and below national poverty lines)

			National			<=5Km			>5Km		
						Malawi					
**Fish consumption by:**	**Fish consumption**	**Indicator**	**Non-poor**	**Poor**	**Total**	**Non-poor**	**Poor**	**Total**	**Non-poor**	**Poor**	**Total**
	Total fish	% of total household	0.82***	0.63***	0.73	0.93***	0.85***	0.89	0.81***	0.60***	0.72
	Fresh fish	% of total household who consumed fish	0.32***	0.22***	0.28	0.75*	0.70*	0.73	0.27***	0.13***	0.22
	Dried fish	% of total household who consumed fish	0.71	0.71	0.71	0.52**	0.45**	0.49	0.73**	0.76**	0.74
Form	Other fish	% of total household who consumed fish	0.32***	0.22***	0.28	0.22**	0.17**	0.2	0.34***	0.23***	0.3
	Total fish	Kg. per household /per week	1.08***	0.62***	0.9	1.83***	1.31***	1.59	0.98***	0.50***	0.8
	Fresh fish	Kg. per household /per week ****	1.54***	1.11***	1.42	1.70***	1.29***	1.53	1.48***	0.94***	1.36
	Dried fish	Kg. per household /per week ****	0.58***	0.41***	0.51	0.77	0.66	0.73	0.56***	0.38***	0.49
	Other fish	Kg. per household /per week ****	0.53***	0.40***	0.49	0.63	0.55	0.6	0.52***	0.38***	0.48
	From purchased	Kg. per household /per week ****	1.03***	0.57***	0.85	1.44***	1.10***	1.29	0.98***	0.48***	0.79
	From own production	Kg. per household /per week ****	3.18**	2.26**	2.77	3.82**	2.47**	3.27	1.91	1.99	1.95
Source	From other source (including gift)	Kg. per household /per week ****	1.40**	0.98**	1.23	2.82***	1.58***	2.26	0.64	0.56	0.61
	From purchased	% of total fish consumed	0.96	0.95	0.96	0.86	0.86	0.86	0.97	0.97	0.97
	From own production	% of total fish consumed	0.01	0.01	0.01	0.06	0.05	0.05	0.00**	0.01**	0
	From other source (including gift)	% of total fish consumed	0.03	0.03	0.03	0.08	0.09	0.09	0.02	0.02	0.02
						Tanzania					
	**Total fish**	**% of total household**	**0.76*****	**0.65*** **	**0.71**	**0.84****	**0.79****	**0.82**	**0.74*** **	**0.62*** **	**0.68**
	Fresh fish	% of total household who consumed fish	0.73*	0.69*	0.71	0.87	0.87	0.87	0.68	0.65	0.67
	Dried fish	% of total household who consumed fish	0.47	0.45	0.46	0.35	0.37	0.36	0.51	0.47	0.49
	Other fish	% of total household who consumed fish	0	0	0	0	0	0	0	0	0
Form	Total fish	Kg. per household /per week	1.06***	0.88***	0.98	1.77	1.91	1.82	0.81***	0.61***	0.73
	Fresh fish	Kg. per household /per week ****	1.09***	0.91***	1.02	1.71	1.87	1.77	0.81***	0.57***	0.71
	Dried fish	Kg. per household /per week ****	0.57	0.55	0.56	0.75	0.76	0.76	0.53	0.51	0.52
	Other fish	Kg. per household /per week ****	0.5	1.5	0.81						
	From purchased	Kg. per household /per week ****	0.97***	0.79***	0.9	1.55	1.69	1.6	0.79***	0.58***	0.7
	From own production	Kg. per household /per week ****	3.73	3.21	3.5	4.66	3.64	4.16	2.66	2.4	2.56
	From other source (including gift)	Kg. per household /per week ****	2.30**	1.52**	1.86	3.38***	1.97***	2.56	1.23	1.01	1.11
Source	From purchased	% of total fish consumed	0.96***	0.94***	0.95	0.92***	0.84***	0.89	0.98	0.96	0.97
	From own production	% of total fish consumed	0.01	0.01	0.01	0.02	0.04	0.03	0.01	0	0.01
	From other source (including gift)	% of total fish consumed	0.03***	0.05***	0.04	0.05***	0.12***	0.08	0.02*	0.03*	0.02
						Uganda					
	**Total fish**	**% of total household**	**0.36*** **	**0.24*** **	**0.34**	**0.49****	**0.37****	**0.46**	**0.34*** **	**0.21*** **	**0.31**
	Fresh fish	% of total household who consumed fish	0.52***	0.40***	0.51	0.74	0.85	0.76	0.47***	0.26***	0.44
	Dried fish	% of total household who consumed fish	0.64	0.67	0.64	0.46**	0.26**	0.42	0.68**	0.80**	0.7
	Other fish	% of total household who consumed fish	0	0	0	0	0	0	0	0	0
Form	Total fish	Kg. per household /per week	1.57***	0.93***	1.47	1.64***	0.77***	1.49	1.55***	0.98***	1.47
	Fresh fish	Kg. per household /per week ****	0.93***	0.55***	0.89	1.09**	0.69**	1.01	0.87***	0.41***	0.83

	Dried fish	Kg. per household /per week ****	1.69***	1.06***	1.6	1.83**	0.68**	1.71	1.67***	1.10***	1.58
	Other fish	Kg. per household /per week ****									
	From purchased	Kg. per household /per week ****	1.54***	0.92***	1.45	1.52**	0.77**	1.38	1.54***	0.97***	1.47
Source	From own production	Kg. per household /per week ****	1.71	0.45	1.59	1.83	0.22	1.77	1.43	0.54	1.24
	From other source (including gift)	Kg. per household /per week ****	1.54	1.18	1.49	1.68	0.44	1.61	1.46	1.28	1.43
	From purchased	% of total fish consumed	0.92	0.93	0.92	0.81**	0.97**	0.84	0.94	0.92	0.94
	From own production	% of total fish consumed	0.03	0.02	0.03	0.10**	0.00**	0.09	0.01	0.02	0.01
	From other source (including gift)	% of total fish consumed	0.05	0.06	0.05	0.09	0.03	0.08	0.05	0.06	0.05
All countries											
**Form**	**Total fish**	**% of total household**	**0.63*****	**0.59*****	**0.62**	**0.75**	**0.74**	**0.75**	**0.61*****	**0.56*****	**0.59**
	Fresh fish	% of total household who consumed fish	0.59***	0.56***	0.58	0.83	0.83	0.83	0.53***	0.49***	0.52
	Dried fish	% of total household who consumed fish	0.56***	0.53***	0.55	0.39	0.38	0.39	0.60***	0.56***	0.59
	Other fish	% of total household who consumed fish	0.07***	0.06***	0.07	0.03	0.04	0.03	0.09***	0.06***	0.08
	Total fish	Kg. per household /per week	1.16***	0.82***	1.04	1.75	1.71	1.74	1.00***	0.60***	0.86
	Fresh fish	Kg. per household /per week ****	1.12***	0.91***	1.05	1.61	1.69	1.64	0.91***	0.59***	0.8
	Dried fish	Kg. per household /per week ****	0.82***	0.54***	0.72	0.98***	0.74***	0.9	0.80***	0.51***	0.69
	Other fish	Kg. per household /per week ****	0.53***	0.40***	0.49	0.63	0.58	0.61	0.52***	0.38***	0.48
	From purchased	Kg. per household /per week ****	1.09***	0.74***	0.97	1.53	1.5	1.52	0.98***	0.57***	0.83
Source	From own production	Kg. per household /per week ****	2.82	2.74	2.8	3.24	3.24	3.24	2.14	2.01	2.09
	From other source (including gift)	Kg. per household /per week ****	1.86***	1.39***	1.65	2.83***	1.88***	2.37	1.16*	0.94*	1.07
	From purchased	% of total fish consumed	0.95***	0.94***	0.95	0.89***	0.85***	0.88	0.97	0.96	0.97
	From own production	% of total fish consumed	0.01	0.01	0.01	0.04	0.04	0.04	0.01	0.01	0.01
	From other source (including gift)	% of total fish consumed	0.03***	0.05***	0.04	0.06***	0.11***	0.08	0.02*	0.03*	0.03

* significant at 10%; ** significant at 5%; *** significant at 1%

Kg. per household per week : average based on households who reported to consume fish

**** averaged among households who reported to consume a given form or source of fish. 0 are not included in the calculation

value is missing if insufficient observations for statistically significant average

Supplementary Table 5 Fish consumption - quantity - by quintile and proximity to water bodies

			National			Rural			Urban		
		Quintile	>5 Km	<=5Km	Total	>5 Km	<=5Km	Total	>5 Km	<=5Km	Total
Fish consumption	Indicator					Malawi					
Total fish	Kg. per household /per week	First	0.42***	1.18***	0.54***	0.42***	1.18***	0.53***	0.56***	1.26	0.69***
Total fish	Kg. per household /per week	Second	0.50***	1.39**	0.63***	0.47***	1.42*	0.60***	0.84***	1.15	0.91***
Total fish	Kg. per household /per week	Third	0.69***	1.57	0.82***	0.67*	1.61	0.81	0.80***	1.31	0.87***
Total fish	Kg. per household /per week	Fourth	0.86**	1.99***	1.01***	0.73***	2.05***	0.92***	1.22*	1.68	1.26
Total fish	Kg. per household /per week	Fifth	1.27***	1.82*	1.31***	0.88***	1.87*	1.00***	1.61***	1.7	1.61***
**Total fish**	**Kg. per household /per week**	**Total**	**0.8**	**1.59**	**0.9**	**0.62**	**1.62**	**0.77**	**1.34**	**1.47**	**1.35**
						Tanzania					
Total fish	Kg. per household /per week	First	0.53***	1.37***	0.69***	0.53***	1.44***	0.68***	0.49	1.1	0.82
Total fish	Kg. per household /per week	Second	0.65**	2.11**	0.95	0.67	2.11	0.94	0.49***	2.11*	1.01
Total fish	Kg. per household /per week	Third	0.77	2.34***	1.12***	0.80*	2.54**	1.13***	0.68	2.01**	1.1
Total fish	Kg. per household /per week	Fourth	0.83***	1.89	1.08**	0.86**	2.49*	1.15***	0.79	1.54	1.01
Total fish	Kg. per household /per week	Fifth	0.84***	1.43***	1.01	0.84	1.49	0.94	0.84**	1.42*	1.04
**Total fish**	**Kg. per household /per week**	**Total**	**0.73**	**1.82**	**0.98**	**0.71**	**2.09**	**0.96**	**0.76**	**1.57**	**1.03**
						Uganda					
Total fish	Kg. per household /per week	First	0.99***	0.78**	0.94***	0.95***	0.78**	0.90***	1.26	0.85	1.2
Total fish	Kg. per household /per week	Second	1.21**	1.08	1.19***	1.24*	1.09	1.21**	0.94*	0.8	0.93*
Total fish	Kg. per household /per week	Third	1.5	1.16	1.42	1.53	1.23	1.45	1.4	0.86	1.29
Total fish	Kg. per household /per week	Fourth	1.51	2.42***	1.66**	1.56	2.86***	1.80***	1.42	0.93	1.37
Total fish	Kg. per household /per week	Fifth	1.87***	1.84	1.86***	2.14***	1.95	2.10***	1.72**	1.75**	1.73***
**Total fish**	**Kg. per household /per week**	**Total**	**1.47**	**1.49**	**1.47**	**1.45**	**1.53**	**1.46**	**1.52**	**1.36**	**1.5**
						All countries					
Total fish	Kg. per household /per week	First	0.56***	1.24***	0.69***	0.55***	1.27***	0.67***	0.73**	1.09*	0.88**
Total fish	Kg. per household /per week	Second	0.69***	1.84	0.91***	0.70***	1.82	0.90***	0.62***	1.92*	0.98**
Total fish	Kg. per household /per week	Third	0.85	2.01***	1.09**	0.87***	2.07**	1.10***	0.80***	1.85**	1.09
Total fish	Kg. per household /per week	Fourth	0.95***	1.98***	1.16***	0.95***	2.45***	1.20***	0.95	1.51	1.09
Total fish	Kg. per household /per week	Fifth	1.13***	1.52***	1.22***	1.07***	1.73	1.17***	1.17***	1.46	1.25***
Total fish	Kg. per household /per week	Total	0.86	1.74	1.04	0.79	1.88	0.98	1	1.54	1.15

Supplementary Table 6 Fishing HHs vs non-fishing HHs - fish consumption, dietary diversity and multiple poverty indicators for Malawi

		All Households			Fishing Households			Non-fishing Households		
					Malawi: National					
**Indicator**	**Measure**	**>5 Km.**	**<=5 Km**	**Total**	**>5 Km.**	**<=5 Km**	**Total**	**>5 Km.**	**<=5 Km**	**Total**
Fish consumption	Kg. per household /per week	0.80***	1.59***	0.9	1.40***	3.36***	2.37	0.79***	1.38***	0.86
Fish consumption from own-production	% of total fish consumed	0.00***	0.05***	0.01	0.17***	0.41***	0.29	0.00***	0.01***	0
Poor household	% of total households	0.44	0.46	0.45	0.37	0.42	0.39	0.45	0.47	0.45
Wealth index	Score (PCA)	1.46	1.51	1.47	1.67	1.46	1.58	1.46	1.51	1.46
Food insecurity	Total months hh experienced food insecuri	3.11*	3.28*	3.13	3.02	2.63	2.84	3.11**	3.35**	3.14
Household with an acceptable FC profile	% of total household	0.62***	0.73***	0.63	0.68**	0.79**	0.73	0.62***	0.73***	0.63
Education of the head of the household	% with no education	0.69***	0.73***	0.7	0.65**	0.77**	0.71	0.69***	0.73***	0.7
Education of the head of the household	% with primary education	0.19	0.19	0.19	0.25	0.2	0.23	0.19	0.19	0.19
Education of the head of the household	% with secondary education	0.10***	0.07***	0.09	0.08**	0.03**	0.06	0.10***	0.07***	0.1
Education of the head of the household	% with tertiary education	0.02**	0.01**	0.02	0.01*	0.00*	0.01	0.02*	0.01*	0.02
Distance to nearest agricultural markets	Km.	21.76***	27.42***	22.4	22.11***	33.99***	27.5	21.75***	26.70***	22.2
Distance to nearest water bodies	Km.	44.63***	2.04***	40.1	36.25***	1.89***	20.6	44.75***	2.05***	40.5
Households with owned or cultivated land	%	0.79	0.78	0.79	0.79***	0.56***	0.69	0.79	0.8	0.79
					Malawi: Rural					
Fish consumption	Kg. per household /per week	0.62***	1.62***	0.77	1.15***	3.33***	2.33	0.61***	1.39***	0.72
Fish consumption from own-production	% of total fish consumed	0.01***	0.06***	0.01	0.21***	0.42***	0.33	0.00***	0.01***	0
Poor household	% of total households	0.52**	0.49**	0.52	0.47	0.43	0.45	0.52	0.5	0.52
Wealth index	Score (PCA)	1.17***	1.33***	1.19	1.36	1.3	1.33	1.16***	1.34***	1.18
Food insecurity	Total months hh experienced food insecuri	3.53**	3.29**	3.5	3.64***	2.57***	3.1	3.53	3.37	3.51
Household with an acceptable FC profile	% of total household	0.55***	0.72***	0.57	0.59***	0.79***	0.69	0.55***	0.71***	0.56
Education of the head of the household	% with no education	0.77	0.76	0.77	0.75	0.76	0.76	0.77	0.76	0.77
Education of the head of the household	% with primary education	0.17	0.18	0.17	0.24	0.21	0.22	0.17	0.18	0.17
Education of the head of the household	% with secondary education	0.05	0.05	0.05	0.01	0.03	0.02	0.05	0.06	0.05
Education of the head of the household	% with tertiary education	0	0	0	0	0	0	0	0	0
Distance to nearest agricultural markets	Km.	25.16***	30.83***	25.8	26.71***	35.67***	31.2	25.13***	30.26***	25.7
Distance to nearest water bodies	Km.	42.84***	1.89***	38.2	29.23***	1.88***	15.6	43.03***	1.89***	38.7
Households with owned or cultivated land	%	0.91***	0.80***	0.9	0.91***	0.55***	0.73	0.91***	0.82***	0.9
					Malawi: Urban					
Fish consumption	Kg. per household /per week	1.34	1.47	1.35	2.15**	3.66**	2.57	1.33	1.3	1.33
Fish consumption from own-production	% of total fish consumed	0.00***	0.03***	0	0.04***	0.31***	0.11	0	0	0
Poor household	% of total households	0.13***	0.31***	0.14	0.03***	0.35***	0.11	0.13***	0.31***	0.14
Wealth index	Score (PCA)	2.66	2.62	2.66	2.71	3.03	2.79	2.66	2.59	2.65
Food insecurity	Total months hh experienced food insecuri	1.43***	3.25***	1.57	0.97***	3.24***	1.54	1.43***	3.25***	1.57
Household with an acceptable FC profile	% of total household	0.91***	0.81***	0.91	1.00***	0.77***	0.94	0.91***	0.82***	0.9
Education of the head of the household	% with no education	0.37***	0.58***	0.38	0.32***	0.87***	0.46	0.37***	0.56***	0.38
Education of the head of the household	% with primary education	0.28*	0.22*	0.28	0.29	0.12	0.25	0.28	0.23	0.28
Education of the head of the household	% with secondary education	0.28***	0.17***	0.27	0.33**	0.01**	0.25	0.28***	0.18***	0.27

Education of the head of the household	% with tertiary education	0.07*	0.03*	0.07	0.06	0	0.05	0.07*	0.03*	0.07
Distance to nearest agricultural markets	Km.	7.92***	5.86***	7.76	6.82***	17.50***	9.51	7.93***	5.02***	7.72
Distance to nearest water bodies	Km.	51.88***	2.96***	48.1	59.61***	2.06***	45.1	51.75***	3.03***	48.2
Households with owned or cultivated land	%	0.30***	0.65***	0.32	0.40**	0.71**	0.48	0.29***	0.65***	0.32

* significant at 10%; ** significant at 5%; *** significant at 1%

Kg. per household per week : average based on households who reported to consume fish

value is missing if insufficient observations for statistically significant average

Supplementary Table 6 Fishing HHs vs non-fishing HHs - fish consumption, dietary diversity and multiple poverty indicators for Tanzania

		All Households			Fishing Households			Non-fishing Households		
						**Tanzania: National**				
**Indicator**	**Measure**	**>5 Km.**	**<=5 Km**	**Total**	**>5 Km.**	**<=5 Km**	**Total**	**>5 Km.**	**<=5 Km**	**Total**
Fish consumption	Kg. per household /per week	0.73***	1.82***	0.98	1.36***	2.99***	2.24	0.71***	1.65***	0.91
Fish consumption from own-production	% of total fish consumed	0.01***	0.03***	0.01	0.17	0.18	0.18	0.00***	0.01***	0
Poor household	% of total households	0.46***	0.37***	0.44	0.4	0.4	0.4	0.46***	0.37***	0.45
Wealth index	Score (PCA)	54.2	57.78	54.9	70.3	60.02	65	53.78	57.51	54.5
Food insecurity	Total months hh experienced food insecuri	1.46	1.37	1.44	1.13	1.45	1.3	1.47	1.36	1.45
Household with an acceptable FC profile	% of total household	0.87***	0.94***	0.89	0.95	0.99	0.97	0.87***	0.93***	0.88
Education of the head of the household	% with no education	0.24**	0.20**	0.23	0.18	0.15	0.16	0.24*	0.21*	0.24
Education of the head of the household	% with primary education	0.61**	0.56**	0.6	0.71	0.77	0.74	0.60***	0.53***	0.59
Education of the head of the household	% with secondary education	0.14***	0.21***	0.16	0.11	0.08	0.1	0.14***	0.23***	0.16
Education of the head of the household	% with tertiary education	0.01***	0.02***	0.01	0	0	0	0.01***	0.03***	0.01
Distance to nearest agricultural markets	Km.	N.A.	N.A.	N.A.	N.A.	N.A.	N.A.	N.A.	N.A.	N.A.
Distance to nearest water bodies	Km.	41.85***	2.19***	33.8	34.99***	1.36***	17.5	42.02***	2.29***	34.5
Households with owned or cultivated land	%	0.75***	0.52***	0.7	0.81**	0.61**	0.71	0.75***	0.50***	0.7
						**Tanzania: Rural**				
Fish consumption	Kg. per household /per week	0.71***	2.09***	0.96	1.42***	2.86***	2.06	0.68***	1.93***	0.88
Fish consumption from own-production	% of total fish consumed	0.01***	0.04***	0.01	0.17	0.16	0.17	0.00***	0.01***	0
Poor household	% of total households	0.59	0.6	0.59	0.45	0.51	0.48	0.6	0.62	0.6
Wealth index	Score (PCA)	44.63	45.67	44.8	58.35	59.67	58.9	44.19	43.44	44.1
Food insecurity	Total months hh experienced food insecuri	1.55	1.58	1.55	1.28	1.55	1.4	1.56	1.58	1.56
Household with an acceptable FC profile	% of total household	0.85***	0.92***	0.86	0.95	0.98	0.97	0.85**	0.91**	0.86
Education of the head of the household	% with no education	0.29	0.28	0.28	0.21	0.12	0.17	0.29	0.3	0.29
Education of the head of the household	% with primary education	0.63	0.62	0.63	0.71	0.79	0.75	0.63	0.59	0.62
Education of the head of the household	% with secondary education	0.08	0.1	0.08	0.08	0.09	0.08	0.08	0.1	0.08
Education of the head of the household	% with tertiary education	0	0	0	0	0	0	0	0	0
Distance to nearest agricultural markets	Km.	N.A.	N.A.	N.A.	N.A.	N.A.	N.A.	N.A.	N.A.	N.A.
Distance to nearest water bodies	Km.	44.38***	2.06***	38.1	34.89***	1.32***	20.4	44.69***	2.17***	39
Households with owned or cultivated land	%	0.91***	0.83***	0.9	0.86	0.79	0.83	0.91***	0.83***	0.9
						**Tanzania: Urban**				
Fish consumption	Kg. per household /per week	0.76***	1.57***	1.03	0.89*	3.22*	2.76	0.76***	1.40***	0.96
Fish consumption from own-production	% of total fish consumed	0.00***	0.02***	0.01	0.19	0.21	0.2	0	0	0
Poor household	% of total households	0.15	0.15	0.15	0.08	0.21	0.18	0.15	0.14	0.15
Wealth index	Score (PCA)	77.50*	69.28*	75	159.17**	60.59**	82.4	76.65	70.05	74.7
Food insecurity	Total months hh experienced food insecuri	1.24	1.17	1.22	0.00*	1.29*	1.01	1.26	1.16	1.23
Household with an acceptable FC profile	% of total household	0.93*	0.95*	0.93	0.92	1	0.98	0.93	0.95	0.93
Education of the head of the household	% with no education	0.13	0.13	0.13	0	0.19	0.14	0.13	0.13	0.13
Education of the head of the household	% with primary education	0.55*	0.50*	0.53	0.69	0.74	0.73	0.55**	0.48**	0.53
Education of the head of the household	% with secondary education	0.3	0.32	0.3	0.31*	0.08*	0.13	0.3	0.34	0.31

Education of the head of the household	% with tertiary education	0.02**	0.04**	0.03	0	0	0	0.02***	0.05***	0.03
Distance to nearest agricultural markets	Km.	N.A.	N.A.	N.A.	N.A.	N.A.	N.A.	N.A.	N.A.	N.A.
Distance to nearest water bodies	Km.	35.67***	2.32***	25.4	35.70***	1.43***	9	35.67***	2.39***	25.9
Households with owned or cultivated land	%	0.35***	0.22***	0.31	0.39	0.33	0.34	0.35***	0.21***	0.31

* significant at 10%; ** significant at 5%; *** significant at 1%

Kg. per household per week : average based on households who reported to consume fish

value is missing if insufficient observations for statistically significant average

Supplementary Table 6 Fishing HHs vs non-fishing HHs - fish consumption, dietary diversity and multiple poverty indicators for Uganda

		All Households			Fishing Households			Non-fishing Households		
		Uganda: National								
**Indicator**	**Measure**	**>5 Km.**	**<=5 Km**	**Total**	**>5 Km.**	**<=5 Km**	**Total**	**>5 Km.**	**<=5 Km**	**Total**
Fish consumption	Kg. per household /per week	1.47	1.49	1.47	1.74	1.49	1.58	1.46	1.48	1.47
Fish consumption from own-production	% of total fish consumed	0.01***	0.09***	0.03	0.26	0.36	0.32	0.01***	0.03***	0.01
Poor household	% of total households	0.2	0.22	0.21	0.17	0.13	0.14	0.2	0.23	0.21
Wealth index	Score (PCA)	1.54	1.49	1.54	1.62	1.92	1.81	1.54	1.44	1.53
Food insecurity	Total months hh experienced food insecuri	1.42	1.46	1.42	1.09	1.47	1.34	1.42	1.46	1.42
Household with an acceptable FC profile	% of total household	0.89	0.91	0.89	0.96	0.84	0.88	0.89	0.91	0.89
Education of the head of the household	% with no education	0.28	0.25	0.28	0.40***	0.02***	0.16	0.28	0.28	0.28
Education of the head of the household	% with primary education	0.49*	0.54*	0.5	0.41***	0.90***	0.73	0.49	0.49	0.49
Education of the head of the household	% with secondary education	0.17	0.18	0.17	0.17	0.08	0.11	0.17	0.19	0.17
Education of the head of the household	% with tertiary education	0.06**	0.03**	0.06	0.02	0	0.01	0.06*	0.04*	0.06
Distance to nearest agricultural markets	Km.	27.07***	33.96***	28.1	33.82***	49.94***	44.2	27.00***	31.90***	27.7
Distance to nearest water bodies	Km.	32.62***	2.57***	28.2	21.99***	2.00***	9.11	32.74***	2.64***	28.7
Households with owned or cultivated land	%	0.53***	0.41***	0.52	0.49	0.5	0.5	0.53***	0.40***	0.52
		Uganda: Rural								
Fish consumption	Kg. per household /per week	1.44	1.53	1.46	1.92	1.54	1.67	1.43	1.52	1.45
Fish consumption from own-production	% of total fish consumed	0.02***	0.11***	0.04	0.32	0.39	0.37	0.01***	0.04***	0.01
Poor household	% of total households	0.24	0.25	0.24	0.19	0.13	0.15	0.24	0.27	0.24
Wealth index	Score (PCA)	1.4	1.4	1.4	1.51	1.95	1.8	1.4	1.31	1.39
Food insecurity	Total months hh experienced food insecuri	1.64	1.7	1.65	1.28	1.55	1.46	1.65	1.72	1.66
Household with an acceptable FC profile	% of total household	0.9	0.91	0.9	0.95	0.83	0.87	0.9	0.93	0.9
Education of the head of the household	% with no education	0.28	0.24	0.27	0.30***	0.02***	0.11	0.28	0.28	0.28
Education of the head of the household	% with primary education	0.54**	0.61**	0.55	0.48***	0.90***	0.76	0.54	0.56	0.54
Education of the head of the household	% with secondary education	0.14	0.13	0.14	0.2	0.08	0.12	0.14	0.13	0.14
Education of the head of the household	% with tertiary education	0.04*	0.02*	0.03	0.02	0	0.01	0.04	0.02	0.04
Distance to nearest agricultural markets	Km.	30.84***	38.80***	32	33.96***	49.11***	44.1	30.80***	37.03***	31.6
Distance to nearest water bodies	Km.	35.82***	2.55***	31	23.96***	1.96***	9.28	35.97***	2.65***	31.7
Households with owned or cultivated land	%	0.65***	0.54***	0.63	0.57	0.53	0.54	0.65***	0.54***	0.63
		Uganda: Urban								
Fish consumption	Kg. per household /per week	1.52	1.36	1.5				1.53	1.39	1.51
Fish consumption from own-production	% of total fish consumed	0	0	0				0	0	0
Poor household	% of total households	0.1	0.12	0.11				0.1	0.13	0.11
Wealth index	Score (PCA)	1.97	1.75	1.94				1.97	1.76	1.94
Food insecurity	Total months hh experienced food insecuri	0.73	0.8	0.74				0.73	0.81	0.74
Household with an acceptable FC profile	% of total household	0.85	0.88	0.85				0.85	0.88	0.85
Education of the head of the household	% with no education	0.3	0.27	0.29				0.29	0.28	0.29
Education of the head of the household	% with primary education	0.34	0.33	0.33				0.34	0.31	0.33
Education of the head of the household	% with secondary education	0.23*	0.31*	0.24				0.23**	0.32**	0.25

Education of the head of the household	% with tertiary education	0.14	0.08	0.13		0.14	0.09	0.13
Distance to nearest agricultural markets	Km.	15.59**	20.10**	16.3		15.48*	19.03*	16
Distance to nearest water bodies	Km.	22.88***	2.63***	19.8		22.96***	2.63***	19.9
Households with owned or cultivated land	%	0.20***	0.04***	0.17		0.20***	0.04***	0.17

* significant at 10%; ** significant at 5%; *** significant at 1%

Kg. per household per week : average based on households who reported to consume fish

value is missing if insufficient observations for statistically significant average

Supplementary Table 6 Fishing HHs vs non-fishing HHs - fish consumption, dietary diversity and multiple poverty indicators for all countries

		All Households			Fishing Households			Non-fishing Households		
					**All countries: National**					
**Indicator**	**Measure**	**>5 Km.**	**<=5 Km**	**Total**	**>5 Km.**	**<=5 Km**	**Total**	**>5 Km.**	**<=5 Km**	**Total**
Fish consumption	Kg. per household /per week	0.86***	1.74***	1.04	1.42***	2.76***	2.15	0.84***	1.58***	0.98
Fish consumption from own-production	% of total fish consumed	0.01***	0.04***	0.01	0.18*	0.24*	0.22	0.00***	0.01***	0
Poor household	% of total households	0.39***	0.35***	0.38	0.36	0.33	0.35	0.39***	0.35***	0.38
Wealth index	Score (PCA)	28.39***	37.52***	29.9	48.51*	38.97*	43.4	28.00***	37.35***	29.5
Food insecurity	Total months hh experienced food insecuri	1.80***	1.63***	1.77	1.43	1.59	1.52	1.81***	1.64***	1.78
Household with an acceptable FC profile	% of total household	0.82***	0.90***	0.84	0.91	0.93	0.92	0.82***	0.90***	0.83
Education of the head of the household	% with no education	0.35***	0.28***	0.34	0.29***	0.19***	0.24	0.35***	0.29***	0.34
Education of the head of the household	% with primary education	0.49**	0.51**	0.49	0.59***	0.74***	0.67	0.48	0.48	0.48
Education of the head of the household	% with secondary education	0.14***	0.19***	0.15	0.11	0.08	0.09	0.14***	0.20***	0.15
Education of the head of the household	% with tertiary education	0.03	0.02	0.02	0.01	0	0	0.03	0.03	0.03
Distance to nearest agricultural markets	Km.	24.75***	31.66***	25.7	27.91***	44.82***	37.5	24.71***	30.05***	25.3
Distance to nearest water bodies	Km.	39.89***	2.26***	33.6	33.15***	1.58***	16.2	40.02***	2.34***	34.2
Households with owned or cultivated land	%	0.70***	0.52***	0.67	0.75***	0.58***	0.66	0.70***	0.52***	0.67
					**All countries: Rural**					
Fish consumption	Kg. per household /per week	0.79***	1.88***	0.98	1.45***	2.61***	2.03	0.77***	1.73***	0.92
Fish consumption from own-production	% of total fish consumed	0.01***	0.06***	0.02	0.20*	0.26*	0.23	0.00***	0.02***	0
Poor household	% of total households	0.48	0.48	0.48	0.41	0.38	0.4	0.48	0.49	0.48
Wealth index	Score (PCA)	22.45***	24.62***	22.8	41.29*	32.89*	37.1	22.02	23.34	22.2
Food insecurity	Total months hh experienced food insecuri	2.03*	1.93*	2.02	1.62	1.7	1.66	2.04	1.96	2.03
Household with an acceptable FC profile	% of total household	0.80***	0.88***	0.81	0.9	0.91	0.9	0.79***	0.88***	0.8
Education of the head of the household	% with no education	0.40***	0.36***	0.39	0.30***	0.18***	0.24	0.4	0.38	0.4
Education of the head of the household	% with primary education	0.50***	0.54***	0.5	0.61***	0.74***	0.67	0.5	0.51	0.5
Education of the head of the household	% with secondary education	0.09	0.1	0.09	0.09	0.08	0.08	0.09	0.1	0.09
Education of the head of the household	% with tertiary education	0.01**	0.01**	0.01	0	0	0	0.01	0.01	0.01
Distance to nearest agricultural markets	Km.	28.27***	35.72***	29.2	30.50***	44.92***	39.2	28.24***	34.34***	29
Distance to nearest water bodies	Km.	41.63***	2.17***	36.1	32.37***	1.61***	17.1	41.83***	2.26***	36.9
Households with owned or cultivated land	%	0.84***	0.74***	0.82	0.82***	0.67***	0.75	0.84***	0.75***	0.82
					**All countries: Urban**					
Fish consumption	Kg. per household /per week	1.00***	1.54***	1.15	1.25***	3.12***	2.57	1.00***	1.39***	1.1
Fish consumption from own-production	% of total fish consumed	0.00***	0.02***	0.01	0.11	0.2	0.17	0.00*	0.00*	0
Poor household	% of total households	0.13	0.15	0.14	0.05*	0.21*	0.16	0.13	0.15	0.14
Wealth index	Score (PCA)	45.42***	56.41***	48.1	92.62	55.45	67	44.92***	56.48***	47.6
Food insecurity	Total months hh experienced food insecuri	1.14	1.21	1.15	0.25***	1.31***	0.98	1.15	1.2	1.16
Household with an acceptable FC profile	% of total household	0.90***	0.93***	0.91	0.95	0.99	0.98	0.90***	0.93***	0.91
Education of the head of the household	% with no education	0.21***	0.17***	0.2	0.25	0.2	0.22	0.21***	0.17***	0.2
Education of the head of the household	% with primary education	0.45	0.46	0.45	0.47**	0.72**	0.65	0.45	0.44	0.45

Education of the head of the household	% with secondary education	0.28**	0.31**	0.29	0.26***	0.07***	0.13	0.28***	0.33***	0.29
Education of the head of the household	% with tertiary education	0.06	0.05	0.06	0.02	0	0.01	0.06	0.05	0.06
Distance to nearest agricultural markets	Km.	12.66***	16.93***	13.2	16.77***	43.45***	25.2	12.62***	16.02***	13
Distance to nearest water bodies	Km.	34.93***	2.39***	27.1	37.89***	1.51***	12.8	34.90***	2.46***	27.5
Households with owned or cultivated land	%	0.30***	0.21***	0.28	0.33	0.33	0.33	0.30***	0.20***	0.28

* significant at 10%; ** significant at 5%; *** significant at 1%

Kg. per household per week : average based on households who reported to consume fish

value is missing if insufficient observations for statistically significant average

Supplementary Table 7 Dietary diversity (food consumption score) by proximity to water bodies

	National			Rural			Urban		
	>5 Km.	<=5 Km	Total	>5 Km.	<=5 Km	Total	>5 Km.	<=5 Km	Total
				Malawi					
Food consumption score	43.63	43.65	43.63	39.41***	43.15***	39.83	60.83***	46.81***	59.75
Food consumption profile==poor	0.16***	0.10***	0.16	0.20***	0.11***	0.19	0.03	0.05	0.03
Food consumption profile==borderline	0.39	0.38	0.39	0.45***	0.39***	0.45	0.15***	0.33***	0.16
Food consumption profile==acceptable/food secure	0.44***	0.52***	0.45	0.35***	0.50***	0.37	0.82***	0.62***	0.81
Total months hh experienced food insecurity	3.11*	3.28*	3.13	3.53**	3.29**	3.5	1.43***	3.25***	1.57
				Tanzania					
	>5 Km.	<=5 Km	Total	>5 Km.	<=5 Km	Total	>5 Km.	<=5 Km	Total
Food consumption score	56.03***	61.54***	57.15	54.39***	59.10***	55.09	60.03***	63.85***	61.21
Food consumption profile==poor	0.05***	0.02***	0.05	0.06*	0.03*	0.05	0.03	0.02	0.03
Food consumption profile==borderline	0.18***	0.10***	0.17	0.21***	0.10***	0.19	0.11	0.1	0.11
Food consumption profile==acceptable/food secure	0.77***	0.88***	0.79	0.73***	0.87***	0.75	0.85	0.89	0.86
Total months hh experienced food insecurity	1.46	1.37	1.44	1.55	1.58	1.55	1.24	1.17	1.22
				Uganda					
	>5 Km.	<=5 Km	Total	>5 Km.	<=5 Km	Total	>5 Km.	<=5 Km	Total
Food consumption score	58.65	58.13	58.58	57.41	57.68	57.45	62.48	59.41	62.01
Food consumption profile==poor	0.06	0.07	0.06	0.04**	0.07**	0.05	0.11	0.07	0.11
Food consumption profile==borderline	0.13**	0.09**	0.12	0.14**	0.10**	0.14	0.09	0.08	0.09
Food consumption profile==acceptable/food secure	0.81	0.84	0.82	0.82	0.83	0.82	0.8	0.86	0.81
Total months hh experienced food insecurity	1.42	1.46	1.42	1.64	1.7	1.65	0.73	0.8	0.74
				All countries					
	>5 Km.	<=5 Km	Total	>5 Km.	<=5 Km	Total	>5 Km.	<=5 Km	Total
Food consumption score	54.09***	58.48***	54.83	51.76***	55.75***	52.32	60.80**	62.47**	61.2
Food consumption profile==poor	0.08***	0.04***	0.07	0.09***	0.06***	0.08	0.05***	0.03***	0.05
Food consumption profile==borderline	0.21***	0.13***	0.2	0.25***	0.15***	0.23	0.11	0.1	0.11
Food consumption profile==acceptable/food secure	0.71***	0.82***	0.73	0.67***	0.79***	0.68	0.83***	0.87***	0.84
Total months hh experienced food insecurity	1.80***	1.63***	1.77	2.03*	1.93*	2.02	1.14	1.21	1.15

* significant at 10%; ** significant at 5%; *** significant at 1%

Supplementary Table 8 Multiple poverty indicators by proximity to water bodies

		National			Rural			Urban		
		>5 Km.	<=5 Km	Total	>5 Km.	<=5 Km	Total	>5 Km.	<=5 Km	Total
Malawi										
Poor household	% of total househods	0.44	0.46	0.45	0.52**	0.49**	0.52	0.13***	0.31***	0.14
Wealth index	Score (PCA)	1.46	1.51	1.47	1.17***	1.33***	1.19	2.66	2.62	2.66
Per-capita expenditure	International US$	2.93	2.86	2.92	2.08***	2.84***	2.17	6.37	2.97	6.11
Distance to nearest agricultural markets	Km.	21.76***	27.42***	22.37	25.16***	30.83***	25.8	7.92***	5.86***	7.76
Education of the head of the household	% with no education	0.69***	0.73***	0.7	0.77	0.76	0.77	0.37***	0.58***	0.38
Education of the head of the household	% with primary education	0.19	0.19	0.19	0.17	0.18	0.17	0.28*	0.22*	0.28
Education of the head of the household	% with secondary education	0.10***	0.07***	0.09	0.05	0.05	0.05	0.28***	0.17***	0.27
Education of the head of the household	% with tertiary education	0.02**	0.01**	0.02	0	0	0	0.07*	0.03*	0.07
Households with cultivated and/or owned land	% of total households	0.79	0.78	0.79	0.91***	0.80***	0.9	0.30***	0.65***	0.32
Tanzania										
Poor household	% of total househods	0.46***	0.37***	0.44	0.59	0.6	0.59	0.15	0.15	0.15
Wealth index	Score (PCA)	54.2	57.78	54.93	44.63	45.67	44.79	77.50*	69.28*	74.96
Per-capita expenditure	International US$	3.70***	4.66***	3.89	2.74	2.58	2.72	6.03**	6.63**	6.22
Distance to nearest agricultural markets	Km.	N.A.	N.A.	N.A.	N.A.	N.A.	N.A.	N.A.	N.A.	N.A.
Education of the head of the household	% with no education	0.24**	0.20**	0.23	0.29	0.28	0.28	0.13	0.13	0.13
Education of the head of the household	% with primary education	0.61**	0.56**	0.6	0.63	0.62	0.63	0.55*	0.50*	0.53
Education of the head of the household	% with secondary education	0.14***	0.21***	0.16	0.08	0.1	0.08	0.3	0.32	0.3
Education of the head of the household	% with tertiary education	0.01***	0.02***	0.01	0	0	0	0.02**	0.04**	0.03
Households with cultivated and/or owned land	% of total households	0.75***	0.52***	0.7	0.91***	0.83***	0.9	0.35***	0.22***	0.31
Uganda										
Poor household	% of total househods	0.2	0.22	0.21	0.24	0.25	0.24	0.1	0.12	0.11
Wealth index	Score (PCA)	1.54	1.49	1.54	1.4	1.4	1.4	1.97	1.75	1.94
Per-capita expenditure	International US$	3.10***	3.74***	3.2	2.47*	2.85*	2.53	5.05**	6.26**	5.23
Distance to nearest agricultural markets	Km.	27.07***	33.96***	28.08	30.84***	38.80***	31.99	15.59**	20.10**	16.27
Education of the head of the household	% with no education	0.28	0.25	0.28	0.28	0.24	0.27	0.3	0.27	0.29
Education of the head of the household	% with primary education	0.49*	0.54*	0.5	0.54**	0.61**	0.55	0.34	0.33	0.33
Education of the head of the household	% with secondary education	0.17	0.18	0.17	0.14	0.13	0.14	0.23*	0.31*	0.24
Education of the head of the household	% with tertiary education	0.06**	0.03**	0.06	0.04*	0.02*	0.03	0.14	0.08	0.13
Households with cultivated and/or owned land	% of total households	0.54***	0.41***	0.52	0.65***	0.54***	0.63	0.20***	0.04***	0.17
All countries										
Poor household	% of total househods	0.39***	0.35***	0.38	0.48	0.48	0.48	0.13	0.15	0.14
Wealth index	Score (PCA)	28.39***	37.52***	29.93	22.45***	24.62***	22.75	45.42***	56.41***	48.07
Per-capita expenditure	International US$	3.37***	4.21***	3.51	2.51	2.71	2.54	5.83	6.42	5.97
Distance to nearest agricultural markets	Km.	24.75***	31.66***	25.65	28.27***	35.72***	29.24	12.66***	16.93***	13.19
Education of the head of the household	% with no education	0.35***	0.28***	0.34	0.40***	0.36***	0.39	0.21***	0.17***	0.2
Education of the head of the household	% with primary education	0.49**	0.51**	0.49	0.50***	0.54***	0.5	0.45	0.46	0.45
Education of the head of the household	% with secondary education	0.14***	0.19***	0.15	0.09	0.1	0.09	0.28**	0.31**	0.29

Education of the head of the household	% with tertiary education	0.03	0.02	0.02	0.01**	0.01**	0.01	0.06	0.05	0.06
Households with cultivated and/or owned land	% of total househods	0.70***	0.52***	0.67	0.84***	0.74***	0.82	0.30***	0.21***	0.28

Supplementary Table 9 Estimated fish consumption - in KG / per capita / year - compared to FAO Supply Data, and scenario for increasing 1% fish supply

	Baseline			
**Country**	**Malawi**	**Tanzania**	**Uganda**	**Total**
Survey Year	2017	2015	2011	
Sample number (households)	12447.0	3352.0	2916.0	18,715.0
Population from the survey	16,307,880	48,152,272	29,345,544	93,805,695
Size(household), weighted stats	4.3	4.7	5.7	5.0
Sample population	53,398	15,888	18,594	87,880
Fish cons.(household/per week)	0.9	1.0	1.5	1.0
Fish cons.(per capita/per week)*	0.21	0.20	0.25	0.2
grams per day	30	29	36	31
Fish cons.(per capita/per year)*, assuming 52.14 week per year	11.0	10.4	13.0	11.1
FAO Fish Supply Data Year	2017	2015	2014	
FAO Fish Supply (kg/capita/year)	9.5	7.2	14.5	
Difference Supply/Consumption	-1.5	-3.2	1.5	

***This is calculated by:**

1 **For each household in the survey, total household fish consumption is divided by the corresponding**

2 **multiplying 1) * 52 weeks (annualized pc fish consumption)**

3 **weighted avearge of 2)**

Supplementary Table 10 Prices of animal source foods purchased by households (adjusted to constant 2010 international US$)

		National			Rural			Urban		
		Malawi								
**Food item**	**Measure**	**>5 Km.**	**<=5 Km**	**Total**	**>5 Km.**	**<=5 Km**	**Total**	**>5 Km.**	**<=5 Km**	**Total**
**Fish**	**Constant 2010 prices per Kg (in international US$)**	**1.39*** **	**1.23*** **	**1.37**	**1.34*** **	**1.18*** **	**1.32**	**1.54**	**1.56**	**1.54**
Fresh fish	Constant 2010 prices per Kg (in international US$)	1.60***	1.24***	1.49	1.32***	1.18***	1.26	1.95**	1.58**	1.9
Dried fish	Constant 2010 prices per Kg (in international US$)	1.34***	1.19***	1.33	1.33***	1.14***	1.31	1.36	1.44	1.37
Other fish	Constant 2010 prices per Kg (in international US$)	1.43***	1.08***	1.4	1.37***	1.03***	1.33	1.55	1.3	1.54
Poultry	Constant 2010 prices per Kg (in international US$)	3.39	2.64	3.37	2.62	2.1	2.57	3.49	3.7	3.49
Goat	Constant 2010 prices per Kg (in international US$)	2.5	2.31	2.49	2.49	2.3	2.48	2.53**	2.35**	2.52
Pork	Constant 2010 prices per Kg (in international US$)	2.63	2.19	2.61	2.68	2.18	2.66	2.47	2.2	2.47
Beef	Constant 2010 prices per Kg (in international US$)	2.61***	2.43***	2.6	2.48	2.51	2.48	2.69***	2.29***	2.67
Eggs	Constant 2010 prices per Kg (in international US$)	2.16	2.14	2.15	2.18	2.21	2.18	2.13**	1.95**	2.12
		Tanzania								
**Fish**	**Constant 2010 prices per Kg (in international US$)**	**4.13*** **	**3.56*** **	**4**	**3.87*** **	**2.85*** **	**3.7**	**4.71*** **	**4.21*** **	**4.55**
Fresh fish	Constant 2010 prices per Kg (in international US$)	4.22***	3.45***	4.02	3.90***	2.66***	3.64	4.82***	4.13***	4.57
Dried fish	Constant 2010 prices per Kg (in international US$)	4.00***	3.48***	3.91	3.85***	2.99***	3.73	4.42	4.18	4.36
Other fish	Constant 2010 prices per Kg (in international US$)									
Poultry	Constant 2010 prices per Kg (in international US$)	6.3	8.22	6.91	6.33	7.52	6.58	6.26	8.58	7.26
Goat	Constant 2010 prices per Kg (in international US$)	5.44	7.18	5.64	5.44	8.07	5.68	5.44	5.67	5.49
Pork	Constant 2010 prices per Kg (in international US$)	4.49	5.08	4.52	4.17	4.02	4.17	5.49	5.53	5.49
Beef	Constant 2010 prices per Kg (in international US$)	5.72	5.7	5.71	5.01	5.7	5.11	6.45	5.71	6.23
Eggs	Constant 2010 prices per Kg (in international US$)	4.57	4.49	4.55	4.61	3.67	4.49	4.55	4.61	4.57
		Uganda								
**Fish**	**Constant 2010 prices per Kg (in international US$)**	**4.29**	**4.17**	**4.26**	**3.94**	**3.96**	**3.95**	**4.84**	**4.63**	**4.81**
Fresh fish	Constant 2010 prices per Kg (in international US$)	4.69	4.29	4.58	4.38	3.99	4.24	5.04	5.59	5.11
Dried fish	Constant 2010 prices per Kg (in international US$)	4.06	4.06	4.06	3.73	3.86	3.74	4.65	4.28	4.59
Other fish	Constant 2010 prices per Kg (in international US$)									
Poultry	Constant 2010 prices per Kg (in international US$)	5.59*	4.02*	5.24	5.82	4.75	5.56	5.45*	3.47*	5.03
Goat	Constant 2010 prices per Kg (in international US$)	5.34	5.34	5.34	5.17	5.29	5.19	5.94	5.72	5.92
Pork	Constant 2010 prices per Kg (in international US$)	4.4	4.68	4.42	4.24	4.54	4.27	5.25	5.83	5.28
Beef	Constant 2010 prices per Kg (in international US$)	5.15	5.21	5.16	4.98	4.99	4.98	5.47	5.63	5.49
Eggs	Constant 2010 prices per Kg (in international US$)	4.11	4.26	4.13	4.3	3.98	4.26	3.93***	4.45***	4.02
		All countries								
**Fish**	**Constant 2010 prices per Kg (in international US$)**	**3.36** **	**3.23** **	**3.34**	**3.09*** **	**2.54*** **	**3**	**3.98**	**4.09**	**4.01**
Fresh fish	Constant 2010 prices per Kg (in international US$)	3.96***	3.21***	3.75	3.67***	2.48***	3.38	4.45***	4.06***	4.32
Dried fish	Constant 2010 prices per Kg (in international US$)	2.99	3.03	3	2.87***	2.52***	2.82	3.33***	3.93***	3.44
Other fish	Constant 2010 prices per Kg (in international US$)	1.43**	1.62**	1.44	1.37***	1.04***	1.33	1.55***	3.64***	1.68
Poultry	Constant 2010 prices per Kg (in international US$)	5.21**	6.58**	5.5	5.82	6.2	5.9	4.83**	6.82**	5.26
Goat	Constant 2010 prices per Kg (in international US$)	4.54***	5.99***	4.69	4.62***	6.31***	4.79	4.25***	5.25***	4.38
Pork	Constant 2010 prices per Kg (in international US$)	3.94**	4.49**	3.98	3.79	4.02	3.8	4.50*	5.33*	4.58
Beef	Constant 2010 prices per Kg (in international US$)	5.34	5.56	5.38	4.89*	5.39*	4.95	5.88	5.65	5.83
Eggs	Constant 2010 prices per Kg (in international US$)	3.46***	3.98***	3.55	3.29*	3.01*	3.26	3.59***	4.39***	3.75

* significant at 10%; ** significant at 5%; *** significant at 1%

value is missing if insufficient observations for statistically significant average

Supplementary Table 11 Variable description

All variables are obtained from the LSMS-ISA household survey, except for proximity to fishing grounds where geospatial data was obtained from the Global Lakes and Wetlands Database (GLWD) (Lehner & Döll, 2004), and the European Space Agency GlobCover databases for coastlines (ESA, 2009).

**Variable**	**Description**
**Demographic**	
Rural households	Household living in rural areas (as defined by the survey)
Urban households	Household living in urban areas (as defined by the survey)
Household size	Total number of household members
Employment	Share of household members in employment over total household members
Age	Age of the head of the household
Male-headed household	Sex of the head of the household
Education of the head of the household: no education, primary, secondary or tertiary	Education level attained by the head of the household: no education, primary, secondary or tertiary.
**Food Environments – physical and economic access to food**	
Proximity to fishing grounds (km)	Distance to water bodies (km) where small-scale fisheries are known to occur; calculated as the shortest distance from the household location to any point of the nearest open water body (km). Anonymity was maintained by applying a 5km buffer around the household locations.
Distance to nearest agriculture market (km)	Distance to agriculture markets
Households unable to reach food market	Households who self-reported to be unable to reach the food market
Livelihoods: small-scale fishing households	Household with at least one member who engaged in small-scale fisheries (fishing, harvesting, processing and/or trading) in the past 12 months. In Uganda only households engaging in harvesting of fish were defined as fishing households, while in Malawi and Tanzania the definition of fishing household extends to also processing and trading.<br>Households defined as fishing households were engaged in small-scale fisheries because the survey excluded commercial businesses, and livelihood activities were characterised by family labour inputs and a small volume of production.
Livelihoods: agriculture households	Household with at least one member who engaged in agriculture (non-fishing) in the past 12 months.
Livelihoods: non-agriculture/fishing households	Household with no members who engaged in agriculture or fishing in the past 12 months.
Average prices (constant 2010 in international US$) of household purchased food item per kg	Price of food item [fish, fresh fish, dried fish, other fish, poultry, goat, pork, beef, eggs] purchased and consumed over past 7 days based on average prices (constant 2010 in international US$) per kg.
**Socio-economic Impacts - poverty**	
Capital assets - wealth index	Total number of assets owned by the household (durable goods - radio, bicycle, TV; utilities and infrastructure – access to protected water source and electricity) with an index developed using principal component analysis
Households with cultivated and/or owned land	Household with land
Per-capita monthly expenditure (local currency unit)	Monthly total expenditure by the household (divided by the total household members)
Share of households living below the national poverty line (local currency unit)	Percentage of households with per-capita monthly expenditure below the national poverty line based on cost of food and non-food basic needs (calculated by national authority) (World Bank, 2020).
**Food Security – diets and seasonality of subjective food insecurity**	
Share (%) of households who consumed animal-source foods over the past 7 days	Percentage of households who self-reported to consume animal-source foods [fish, poultry, goat, pork, beef, eggs] at least once over 7 days.
Share (%) of households who consumed dried, fresh or other form of fish over the past 7 days	Percentage of households who self-reported to consume dried, fresh or other form of fish over 7 days.
Share (%) of households who consumed fish over the past 7 days from purchased, own production or other source (including gifts).	The share of households who obtained fish for consumption over the past 7 days via purchases, own production or other sources, including gifts.
Number of days households consumed fish over the past 7 days	Frequency of fish (general, dried, fresh and other) consumed over 7 days

Quantity of fish (general, dried, fresh and other) consumed over the past 7 days (kg/per week/per-household)	Quantity of fish consumed; general and by form (dried, fresh and other), by the households acquired from purchased, own consumption or gifts
Food consumption score (FCS)	An index capturing food consumption profiles at the household level. Based on the diversity and frequency of food groups consumed over a 7-day recall period,<br>with weights given to groups based on nutritional value. Validated as a proxy for energy sufficiency (quantity of food) (Leroy et al., 2015; World Food Programme, 2008).
Food consumption profile: poor, borderline, acceptable	Household with a food consumption score that is defined according to World Food Programme (2008) universal classification as poor (FCS<28), borderline (FCS>=28 and <41) or acceptable (FCS>=41), where acceptable represents food secure households.
Food insecure household over the past 12 months	Subjective food insecurity defined as households who have experienced a situation in the last 12 months when they did not have enough food to feed the household.
Total months household self-reported food insecurity over the past 12 months	The number of months during over the past 12 months that a household reported not having enough food to feed the household

Supplementary Table 12 Nutrient content of fish species caught from small-scale fisheries in the region and other animal source foods (per 100g edible food). Bold highlights the food items that contain the top three nutrient values for each nutrient. Data source: (FishBase, 2021; FAO et al, forthcoming; MAFOODS, 2019).

Food name / Fish species (with local name)	Calcium (mg)	Iron (mg)	Selenium (mcg)	Zinc (mg)	Vitamin A (mcg, RAE)	Omega_3 (DHA+EPA) (g)	Protein (g)
**Small* freshwater fish (fresh)**							
Rastrineobola argentea (Dagaa)	**836.85**	3.37	32.25	2.78	**82.22**	**0.59**	18.03
Engraulicypris sardella (Usipa)	735.89	7.08	59.24	2.21	49.13	0.32	17.17
Stolothrissa tanganicae (Kapenta)	540.42	4.13	46.37	2.96	25.11	**0.45**	17.91
Copadichromis virginalis (Utaka)	246.02	1.66	45.69	2.5	59.27	0.39	17.99
Protomelas similis	226.11	2.02	52.24	3.08	24.39	0.3	17.61
Limnothrissa miodon (Kapenta)	309.78	4.57	51.9	2.8	10.39	**0.51**	18.09
**Dried small freshwater fish**							
Engraulicypris breianalis (Usipa)	**1453**	6.2	-	**25.4**	66	-	**67.2**
Engraulicypris sardella (Usipa)	451	**40.6**	-	-	-	-	**45.7**
Rhamphochromis esox (Mcheni)	**1884**	**20.3**	-	-	-	-	39.8
Copadichromis inornatus (Utaka)	451	**40.6**	-	-	-	-	**45.7**
**Large* freshwater fish (fresh)**							
Lates niloticus (Nile perch, mbuta)	133.57	1.74	**195.52**	0.94	13.86	0.29	20
Oreochromis niloticus (Nile tilapia)	25.93	1.79	**74.22**	1.91	3.77	0.41	17.34
Oreochromis karongae (Chambo)	19.93	1.79	59.53	2.36	12.29	0.26	17.52
Clarias gariepinus (Catfish, Mlamba)	20.16	1.6	**73.31**	0.55	32.3	0.25	17.34
**Other animal-source foods**							
Beef	1	7.5	-	1.77	-	-	20.5
Beef (liver)	7	8.8	-	**3.5**	**4970**	-	19.4
Goat	11	2.4	-	3.45	0	-	17.5
Pork	10	1.4	-	**3.6**	0	-	16.8
Chicken meat (with skin)	8	1	-	1.6	7	-	21.1
Chicken egg	39	1.8	-	1.15	**67**	-	12.6
Cow's milk	120	0.1	-	0.38	44	-	2.9

***Fresh water fish species (small and large) are those most commonly caught from small-scale fisheries in Malawi, Tanzania and Uganda and are defined as small (maximum length <25cm) and large (maximum length >25cm). Their nutrient values are derived from FishBase and a nutrition modelling study. Data source: (FishBase, 2021; FAO et al., n.d.).

Note: Dried small freshwater fish and other animal-source foods and their nutrient values are derived from Malawi's Food Composition Table. Data source: (MAFOODS, 2019). Due to the different data sources and methods used in calculating the nutritional value of dried fish compared with fresh, no direct comparisons can be made between the two. Note that Engralicypris breianalis is the same species as E. sardella, and taxonomically E. breianalis is a misnomer. In addition, Utaka is a common local name for a mixture of many small pelagic cichlids from Lake Malawi.

FAO, Duke University, & WorldFish. (n.d.). Illuminating Hidden Harvests: The contribution of small-scale fisheries to sustainable development.
FishBase. 2021. FishBase. www.fishbase.org. Accessed June 30, 2021
MAFOODS. (2019). *Malawian Food Composition Table 2019* .

		% of households that consumed:					Price per Kg				
		**Mean**	Std. Err.	Margin of error	[95% Conf.	Interval]	**Mean**	Std. Err.	Margin of error	[95% Conf.	Interval]
Malawi	Fish	**73%**	0.004729	0.009269232	72.55%	74.40%	**1.37**	0.011363	0.022271284	1.3501	1.3946
	Poultry	**13%**	0.003744	0.00733824	12.14%	13.61%	**3.37**	0.082527	0.161753116	3.2114	3.5350
	Goat	**13%**	0.003833	0.007513072	12.69%	14.20%	**2.49**	0.054781	0.107370564	2.3851	2.5999
	Pork	**7%**	0.002855	0.005595996	6.07%	7.19%	**2.61**	0.136818	0.2681623	2.3455	2.8819
	Beef	**9%**	0.003197	0.006265924	8.84%	10.09%	**2.60**	0.01761	0.03451462	2.5671	2.6361
	Eggs	**31%**	0.005114	0.010022656	30.08%	32.09%	**2.15**	0.009829	0.01926484	2.1343	2.1729
Tanzania	Fish	**71%**	0.009424	0.018471432	69.35%	73.05%	**4.00**	0.052562	0.103020736	3.9019	4.1080
	Poultry	**17%**	0.007613	0.014922068	15.41%	18.40%	**6.91**	0.637946	1.250373572	5.6635	8.1649
	Goat	**12%**	0.007072	0.01386112	11.10%	13.87%	**5.64**	0.415386	0.814157148	4.8269	6.4557
	Pork	**4%**	0.00396	0.007762384	3.13%	4.68%	**4.52**	0.134093	0.2628213	4.2618	4.7876
	Beef	**40%**	0.009908	0.0194187	37.92%	41.80%	**5.71**	0.221904	0.434932232	5.2793	6.1494
	Eggs	**20%**	0.008046	0.015769572	18.05%	21.20%	**4.55**	0.164774	0.32295606	4.2262	4.8723
Uganda	Fish	**33%**	0.010765	0.021099008	31.26%	35.48%	**4.26**	0.137479	0.26945786	3.9950	4.5342
	Poultry	**3%**	0.00401	0.007860384	1.91%	3.48%	**5.24**	0.436139	0.854831852	4.3868	6.0975
	Goat	**4%**	0.004623	0.009060884	3.43%	5.24%	**5.34**	0.091639	0.179613224	5.1608	5.5203
	Pork	**6%**	0.005172	0.010137904	4.60%	6.63%	**4.42**	0.080342	0.157469928	4.2650	4.5802
	Beef	**30%**	0.010684	0.020940444	28.30%	32.49%	**5.16**	0.032035	0.062788012	5.0955	5.2211
	Eggs	**9%**	0.007314	0.014334852	7.85%	10.71%	**4.13**	0.056921	0.11156516	4.0233	4.2465

Note: Confidence interval around the mean are calculated as: mean +/- margin of error

The confidence interval indicate that we are 95 % confident that the real mean of each calculated statstics is comprised which is the lower

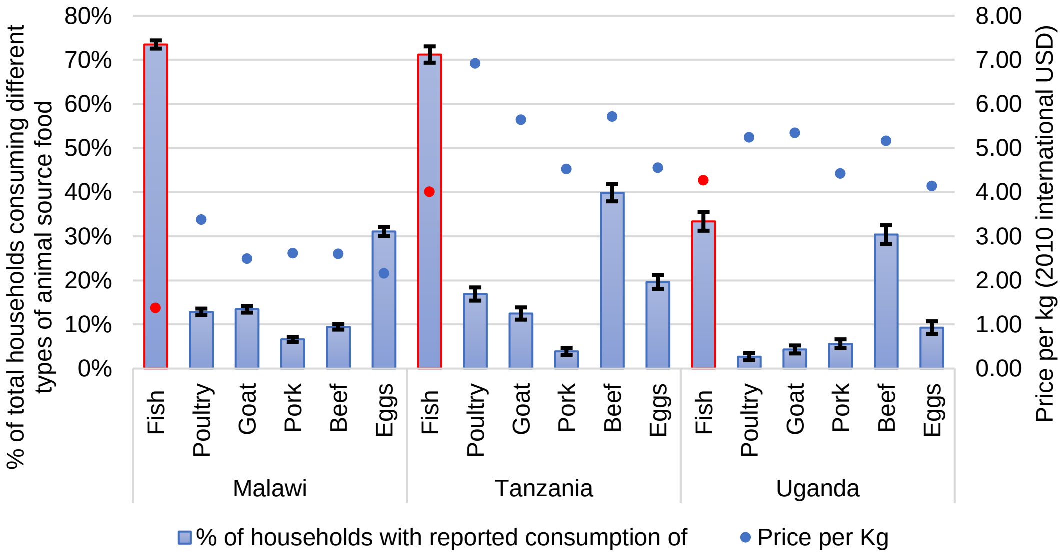

Supplementary Fig 1. Share of households (% of total) consuming animal source foods and prices of purchased food (average price per kilogram in international US$). Red highlights fish**.** Lines show 95 % confidence interval (mean +/-1.96*standard error).

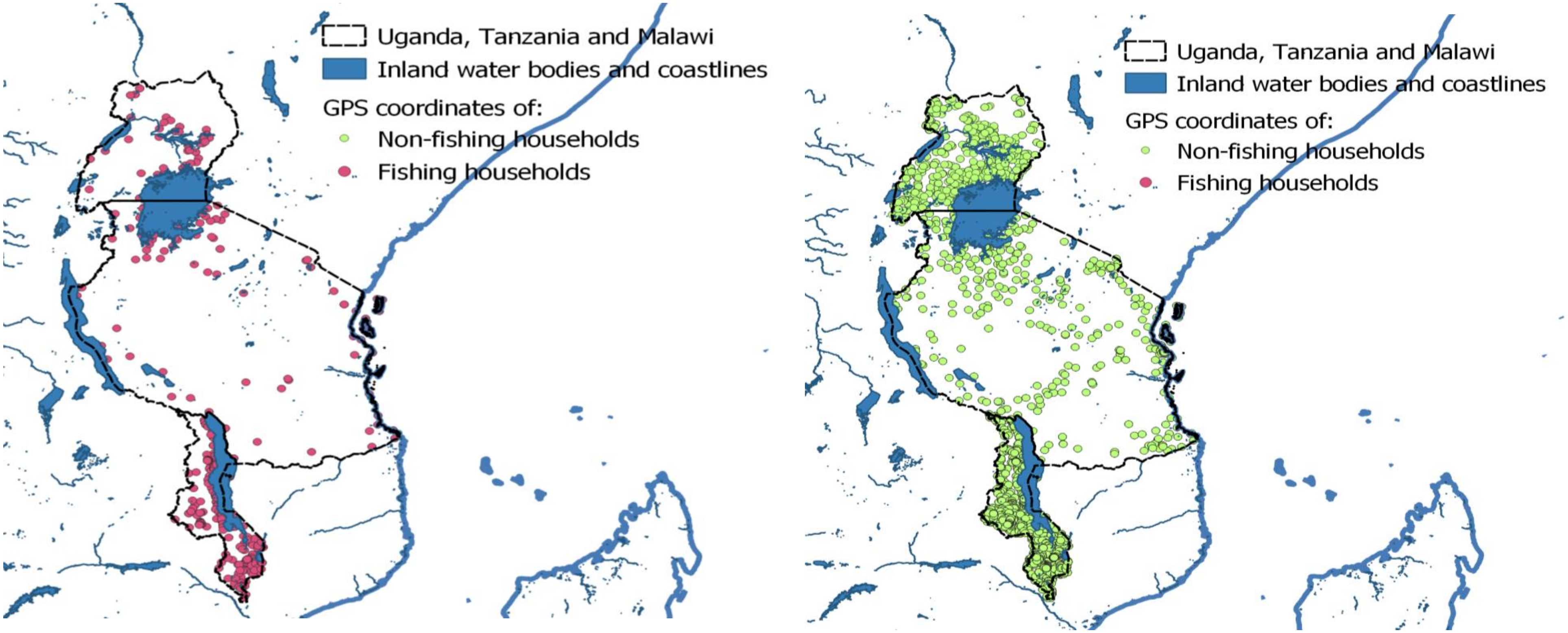

Spatial distribution of fishing households (red dots) and non-fishing households (green dots) by open inland water bodies (≥0.1 km2) and coastlines for Uganda, Tanzania and Malawi.

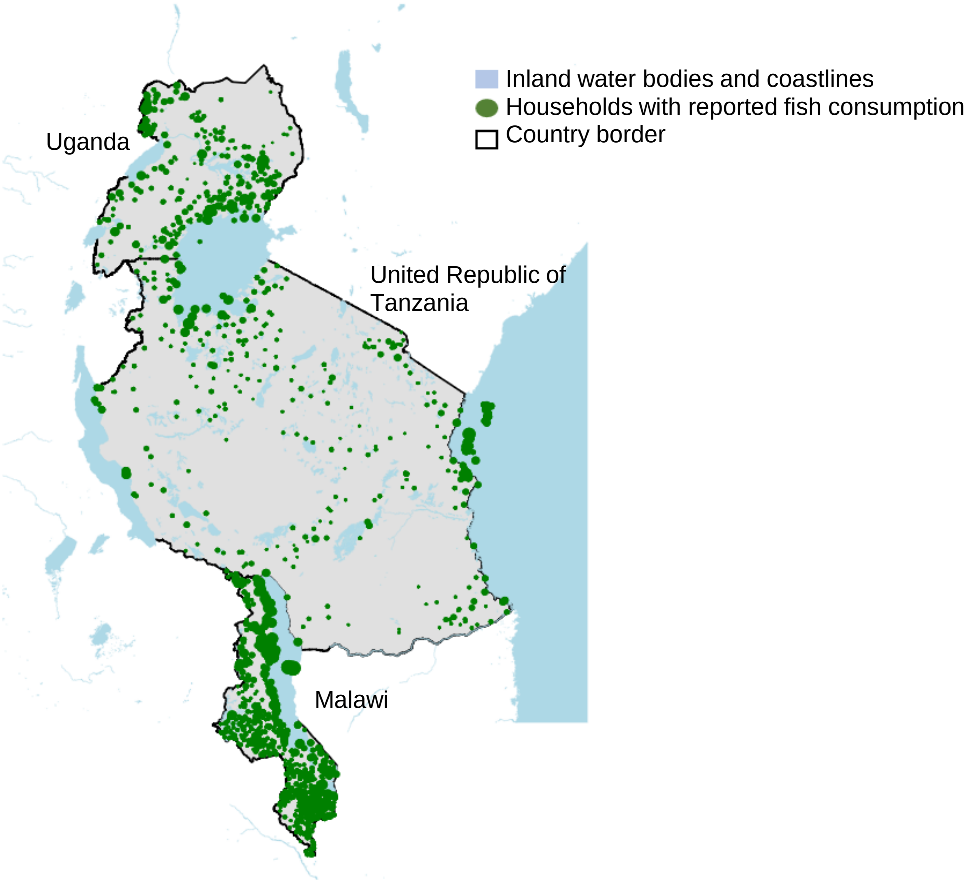

Supplementary Fig 2. Spatial distribution of households reporting any fish consumption (green dots) by open inland water bodies (≥0.1 km2) and coastlines for Uganda, Tanzania and Malawi. Analysis is of 18,715 households and approximately 87,879 people; a sample framed to be representative of the total population of each country (93.8 million population in total for all three countries). Data source: the World Bank's Living Standards Measurement Surveys and Integrated Surveys on Agriculture (LSMS-ISA) for Malawi (2016–17), Tanzania (2014–15) and Uganda (2010–11); Global Lakes and Wetlands Database (GLWD) (Lehner & Döll, 2004), and the European Space Agency GlobCover databases for coastlines (ESA, 2009).

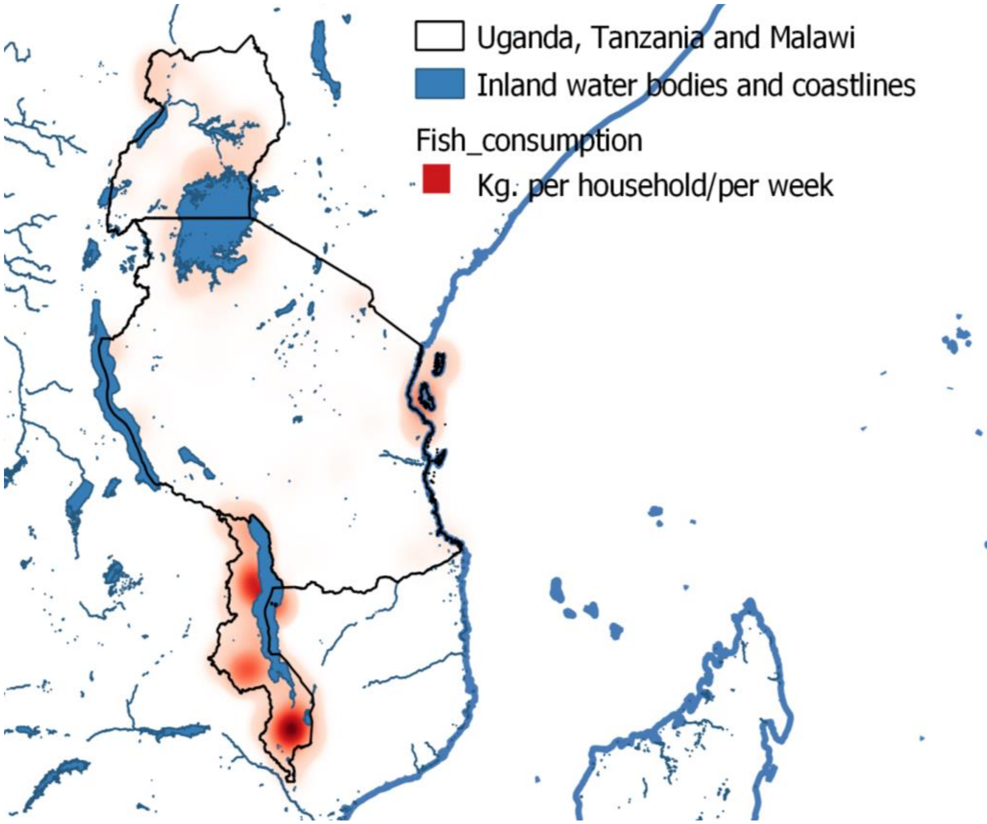

Spatial distribution of fish consumption (kg/household/week) by open inland water bodies (≥0.1 km2) and coastlines for Uganda, Tanzania and Malawi.

	Distance	Quintile	National Mean fish consumption	National Std. Err.	Rural Mean fish consumption	Rural Std. Err.	Urban Mean fish consumption	Urban Std. Err.
Malawi	>5 Km	1	0.42	0.0168263	0.42	0.015819	0.56	0.1545944
		5	1.27	0.0413091	0.88	0.0478636	1.61	0.0625937
	<=5 Km	1	1.18	0.1113746	1.18	0.1133232	1.26	0.5340947
		5	1.82	0.1798429	1.87	0.2289092	1.70	0.2123266
Tanzania	>5 Km	1	0.53	0.0373447	0.53	0.0389429	0.49	0.0645484
		5	0.84	0.0484187	0.84	0.1118638	0.84	0.0508833
	<=5 Km	1	1.37	0.1503724	1.44	0.170413	1.10	0.3123995
		5	1.43	0.113695	1.49	0.197979	1.42	0.1264895
Uganda	>5 Km	1	0.99	0.0903261	0.95	0.08404	1.26	0.3843559
		5	1.87	0.176383	2.14	0.373277	1.72	0.1772149
	<=5 Km	1	0.78	0.1040492	0.78	0.1064047	0.85	0.4708359
		5	1.84	0.2872595	1.95	0.4264442	1.75	0.3863968
All countries	>5 Km	1	0.56	0.027457	0.55	0.0278337	0.73	0.1405518
		5	1.13	0.0414617	1.07	0.082919	1.17	0.0454528
	<=5 Km	1	1.24	0.1028901	1.27	0.1105637	1.09	0.2735713
		5	1.52	0.0978253	1.73	0.1648694	1.46	0.1168233

Malawi graph (3) fish consumption

		Rural		Urban	
		>5km	<=5 Km	>5km	<=5 Km
First	First quintile	0.42	1.18	0.56	1.26
	Std error	0.02	0.11	0.15	0.53
Fifth	Fifth quintile	0.88	1.87	1.61	1.70
	Std error	0.05	0.23	0.06	0.21

Tanzania graph (3) fish consumption

		Rural		Urban	
		>5km	<=5 Km	>5km	<=5 Km
First	First quintile	0.53	1.44	0.49	1.10
	Std error	0.04	0.17	0.06	0.31
Fifth	Fifth quintile	0.84	1.49	0.84	1.42
	Std error	0.11	0.20	0.05	0.13

Uganda graph (3) fish consumption

		Rural		Urban	
		>5km	<=5 Km	>5km	<=5 Km
First	First quintile	0.95	0.78	1.26	0.85
	Std error	0.08	0.11	0.38	0.47
Fifth	Fifth quintile	2.14	1.95	1.72	1.75
	Std error	0.37	0.43	0.18	0.39

Data for Graph 4

Malawi urban >5 km	Uganda rural ≤5 km	Uganda rural >5 km	Malawi rural >5 km	Uganda urban ≤5 km	Tanzania urban >5 km
2.9	2.5	2.3	2.1	2.1	1.7
Uganda urban >5 km	Malawi urban ≤5 km	Tanzania urban ≤5 km	Tanzania rural ≤5 km	Tanzania rural >5 km	Malawi rural ≤5 km
1.4	1.4	1.3	1	1.6	1.6

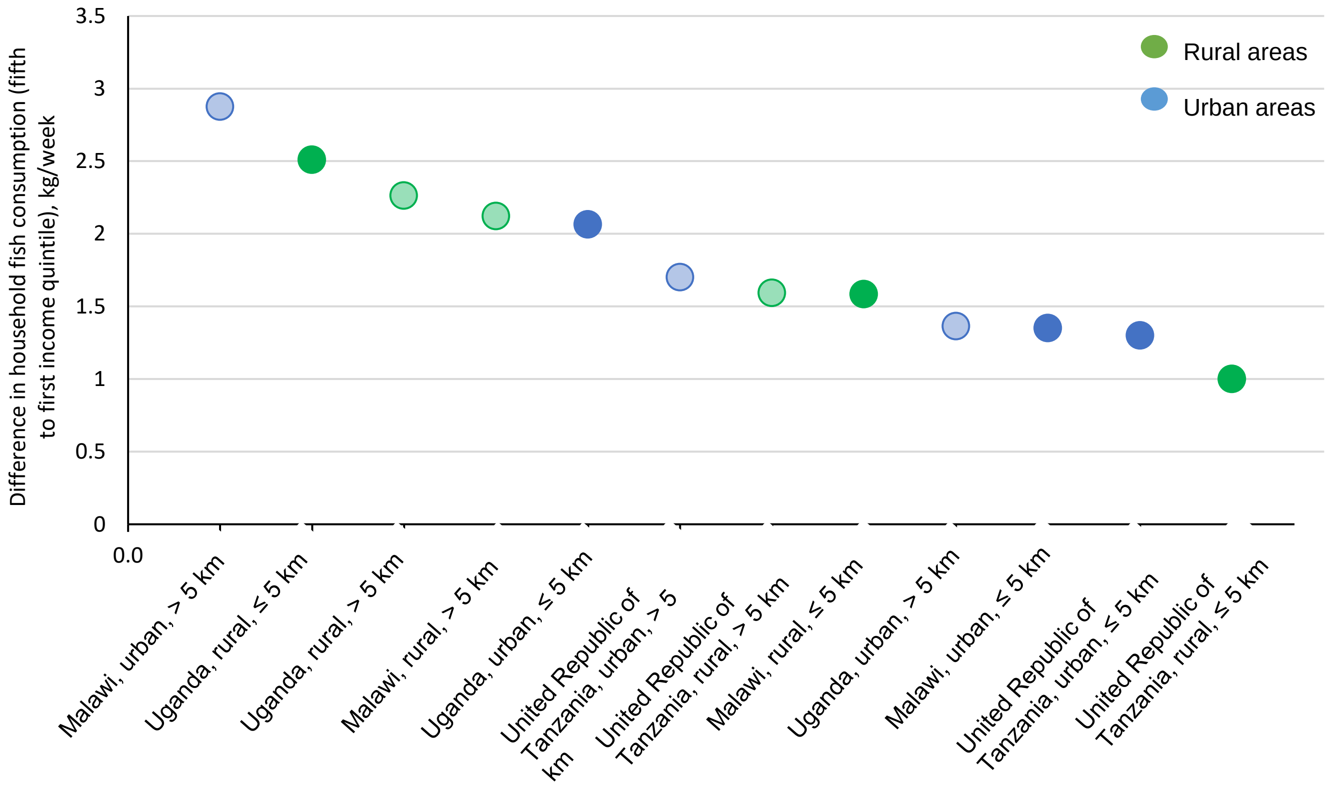

Supplementary Fig 4. Difference in the quantities of fish consumed (kg/household/week) between richest (fifth quintile) and poorest (first quintile) households, by rural and urban areas and proximate (<5km) or distant (>5km) from water bodies in Malawi, Tanzania and Uganda. Graph depicts greater inequalities in consumption of fish in contexts on the left, and lower inequalities on the right. Green represents rural areas, and blue urban, whilst solid colours represent proximate to fishing grounds, and faded distant.

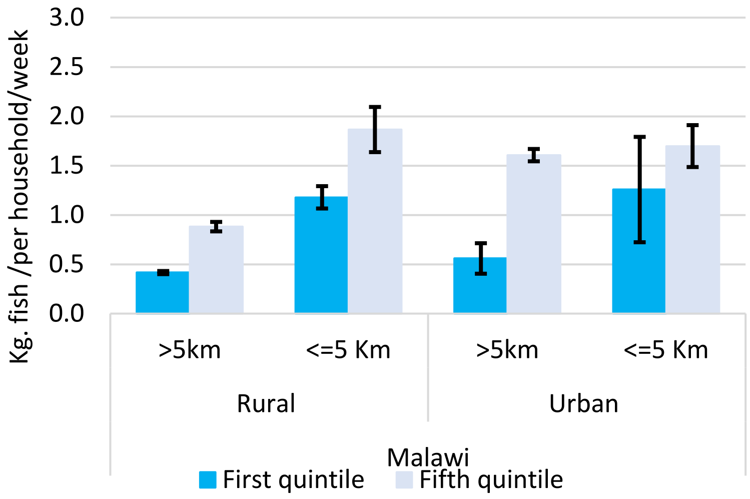

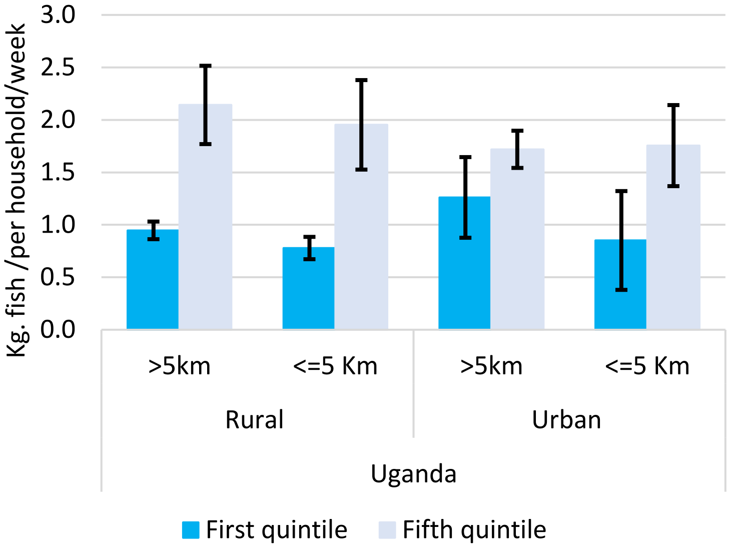

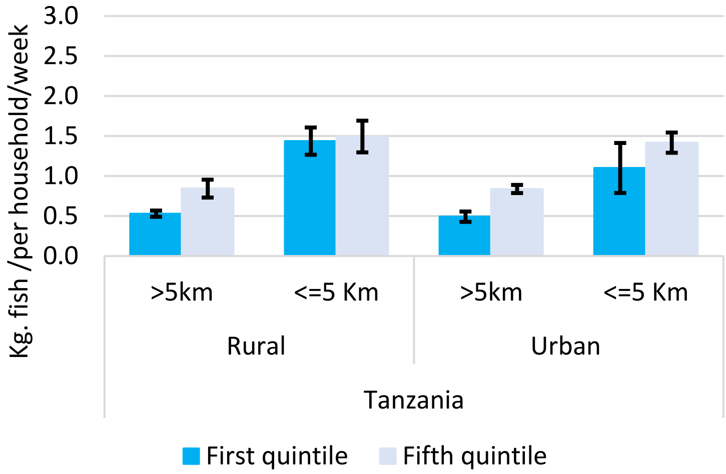

Supplementary Fig 3. Fish consumption (kg/household/week) by richest (fifth quintile) and poorest (first quintile) households, in rural and urban areas that are proximate (<5km) or distant (>5km) from water bodies in Malawi, Tanzania and Uganda.

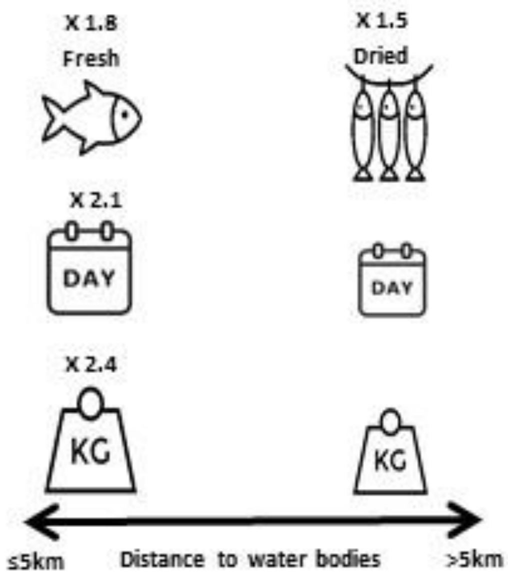

Supplementary Fig 5. Fish consumption by proximity to water bodies across rural Malawi, Tanzania and Uganda. Representing the average difference in the share of total households consuming dried and fresh fish, quantity of fish consumed (kg/household/week) and frequency (number of days out of 7 days).

Malawi fish consumption

		Malawi > 5Km	Malawi <= 5Km
Poor	poor	0.36	0.86
	Std error	0.02	0.19
Borderline	Borderline	0.48	1.09
	Std error	0.02	0.07
Acceptable/food secure	Acceptable/food secure	1.10	2.00
	Std error	0.03	0.09

Tanzania fish consumption

		Tanzania > 5Km	Tanzania <= 5Km
Poor	poor	0.29	0.48
	Std error	0.06	0.14
Borderline	Borderline	0.37	0.69
	Std error	0.03	0.10
Acceptable/food secure	Acceptable/food secure	0.80	1.93
	Std error	0.03	0.09

Uganda fish consumption

		Uganda > 5Km	Uganda <= 5Km
Poor	poor		
	Std error		
Borderline	Borderline	0.83	0.37
	Std error	0.13	0.07
Acceptable/food secure	Acceptable/food secure	1.51	1.55
	Std error	0.06	0.14

All countries fish consumption

		All countries > 5Km	All countries <= 5Km
Poor	poor	0.34	0.65
	Std error	0.02	0.11
Borderline	Borderline	0.45	0.87
	Std error	0.02	0.06
Acceptable/food secure	Acceptable/food secure	0.98	1.88
	Std error	0.02	0.07

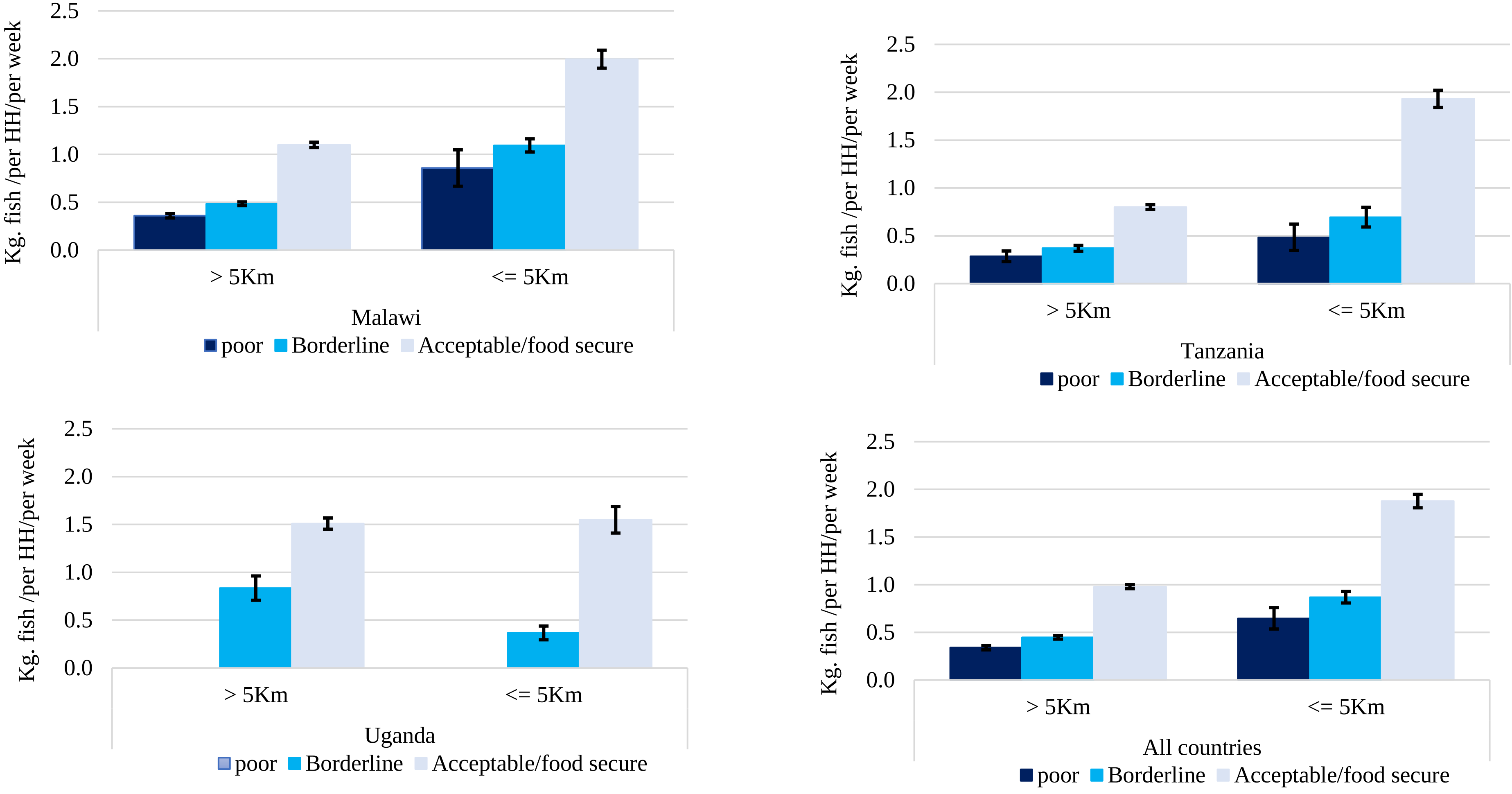

Supplementary Fig 6. Fish consumption (average kg/household/week) by food consumption profile and proximity to water bodies. Note: food consumption profile is based on the household Food Consumption Score (FCS), which measures frequency and diversity of food groups con

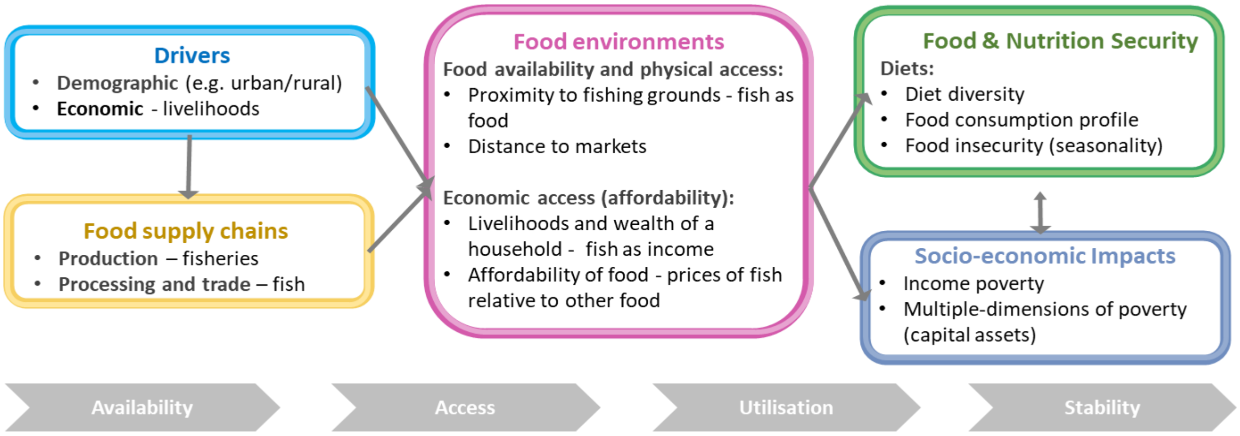

Supplementary Fig 7. Conceptual framework. (Adapted from the Food Systems Framework (HLPE, 2017) with permission). Boxes and arrows represent the components and relationships analysed in the study.
