# Supplementary material for: Proximity to small-scale inland and coastal fisheries is associated with improved income and food security
Source: Commun Earth Environ. 2022 Aug 3;3(1):174. doi: 10.1038/s43247-022-00496-5 (PMC9362682; doi:10.1038/s43247-022-00496-5)
Supplement: Supplementary file 2 — Supplementary Data 1 [file 43247_2022_496_MOESM2_ESM.docx]

**Table 1. Probit regression: estimated marginal effects at the mean distance to water bodies on the probability to be income poor (living below the national poverty line)**

| VARIABLES | All countries | | Malawi | | Tanzania | | Uganda | |
| --- | --- | --- | --- | --- | --- | --- | --- | --- |
|  | National | Rural | National | Rural | National | Rural | National | Rural |
| **Distance to nearest water body (km)** | **0.00136***** | **0.00136***** | **0.00077***** | **0.00061***** | **0.00060***** | **0.00008***** | **0.00164***** | **0.00214***** |
|  | (0.00000) | (0.00001) | (0.00001) | (0.00002) | (0.00001) | (0.00001) | (0.00001) | (0.00001) |
| Distance to nearest water body (km) of households unable to reach food markets | 0.00310*** | 0.00343*** | 0.00931*** | 0.01075*** | -0.00494*** | -0.00419*** | 0.00154*** | 0.00163*** |
|  | (0.00002) | (0.00003) | (0.00019) | (0.00021) | (0.00013) | (0.00013) | (0.00002) | (0.00002) |
| Distance to nearest agricultural market (km) |  |  | -0.00000 | -0.00294*** |  |  | -0.00007*** | -0.00075*** |
|  |  |  | (0.00002) | (0.00003) |  |  | (0.00001) | (0.00001) |
| Neither fishing nor agriculture HHs, close to water bodies | 0.13008*** | 0.19429*** | 0.14556*** | 0.02641*** | 0.03553*** | 0.02682*** | 0.20714*** | 0.33079*** |
|  | (0.00075) | (0.00116) | (0.00295) | (0.00326) | (0.00093) | (0.00171) | (0.00155) | (0.00239) |
| Fishing HHs, close to water bodies | 0.05244*** | 0.06937*** | 0.02854*** | -0.01141* | 0.00896*** | 0.08416*** | 0.03679*** | 0.04943*** |
|  | (0.00128) | (0.00149) | (0.00548) | (0.00609) | (0.00159) | (0.00177) | (0.00246) | (0.00292) |
| Agriculture HHs, close to water bodies | 0.05363*** | 0.04467*** | -0.07513*** | -0.08115*** | 0.02897*** | 0.02980*** | 0.08171*** | 0.10409*** |
|  | (0.00043) | (0.00050) | (0.00103) | (0.00116) | (0.00062) | (0.00073) | (0.00076) | (0.00092) |
| Households unable to reach food markets | -0.08354*** | -0.07506*** | -0.29404*** | -0.37178*** | 0.45665*** | 0.24855*** | 0.02345*** | 0.04840*** |
|  | (0.00098) | (0.00123) | (0.00453) | (0.00552) | (0.00411) | (0.00438) | (0.00086) | (0.00110) |
| Fishing households | 0.15921*** | 0.06537*** | 0.06462*** | -0.08396*** | 0.21707*** | 0.12305*** | 0.05066*** | 0.09241*** |
|  | (0.00086) | (0.00109) | (0.00197) | (0.00251) | (0.00115) | (0.00153) | (0.00187) | (0.00243) |
| Agriculture households | 0.24929*** | 0.17300*** | 0.18251*** | 0.00937*** | 0.35963*** | 0.27708*** | 0.05557*** | 0.05965*** |
|  | (0.00030) | (0.00058) | (0.00082) | (0.00139) | (0.00042) | (0.00093) | (0.00050) | (0.00074) |
| Household consumed fish (past 7 days) | -0.08574*** | -0.07206*** | -0.24685*** | -0.22931*** | -0.12523*** | -0.07070*** | -0.04466*** | -0.05201*** |
|  | (0.00027) | (0.00032) | (0.00065) | (0.00066) | (0.00040) | (0.00045) | (0.00036) | (0.00046) |
| Household size | 0.03653*** | 0.03951*** | 0.11973*** | 0.13133*** | 0.05730*** | 0.07213*** | 0.01881*** | 0.02654*** |
|  | (0.00005) | (0.00006) | (0.00019) | (0.00021) | (0.00008) | (0.00010) | (0.00006) | (0.00008) |
| Ratio employed household member over not employed | -0.13830*** | -0.14823*** | -0.04342*** | -0.06581*** | -0.21565*** | -0.18829*** | -0.10651*** | -0.08681*** |
|  | (0.00044) | (0.00054) | (0.00100) | (0.00108) | (0.00065) | (0.00078) | (0.00070) | (0.00089) |
| Age of the household head | -0.00008* | 0.00172*** | -0.00723*** | -0.00515*** | -0.00380*** | 0.00275*** | -0.00092*** | -0.00193*** |
|  | (0.00004) | (0.00005) | (0.00010) | (0.00011) | (0.00007) | (0.00008) | (0.00007) | (0.00008) |
| Age of the household head, quadratic | -0.00001*** | -0.00003*** | 0.00006*** | 0.00004*** | 0.00001*** | -0.00006*** | 0.00001*** | 0.00003*** |
|  | (0.00000) | (0.00000) | (0.00000) | (0.00000) | (0.00000) | (0.00000) | (0.00000) | (0.00000) |
| Sex of the head of the household | -0.04366*** | -0.05376*** | -0.05509*** | -0.06445*** | -0.08875*** | -0.09490*** | 0.04974*** | 0.03340*** |
|  | (0.00028) | (0.00034) | (0.00067) | (0.00072) | (0.00041) | (0.00048) | (0.00036) | (0.00049) |
| Education of the head of the household - Primary | -0.07831*** | -0.06867*** | -0.15394*** | -0.14060*** | -0.20624*** | -0.15392*** | -0.01452*** | -0.00136** |
|  | (0.00030) | (0.00035) | (0.00072) | (0.00084) | (0.00044) | (0.00048) | (0.00047) | (0.00056) |
| Education of the head of the household - Secondary | -0.31264*** | -0.32828*** | -0.32729*** | -0.31063*** | -0.44218*** | -0.35090*** | -0.09635*** | -0.07422*** |
|  | (0.00034) | (0.00048) | (0.00090) | (0.00138) | (0.00052) | (0.00085) | (0.00056) | (0.00075) |
| Wealth index | -0.00035*** | 0.00060*** | -0.07794*** | -0.07426*** |  | -0.00507*** | -0.11681*** | -0.14009*** |
|  | (0.00000) | (0.00000) | (0.00018) | (0.00020) |  | (0.00001) | (0.00022) | (0.00030) |
| Baseline: average probability to be poor (*100) | 0.384 | 0.478 | 0.446 | 0.517 | 0.443 | 0.593 | 0.212 | 0.239 |
| Distance to nearest water bodies, in Km. | 33.1 | 36.0 | 37.0 | 37.1 | 33.8 | 38.0 | 28.5 | 31.2 |
| Observations | 18,610 | 14,275 | 12,444 | 10,174 | 3,344 | 1,978 | 2,817 | 2,123 |
| Country/district FE | Yes | Yes | Yes | Yes | Yes | Yes | Yes | Yes |
| r2 | 0.1734 | 0.122 | 0.2539 | 0.2118 | 0.2343 | 0.2077 | 0.1815 | 0.1639 |
| Standard errors in parentheses *** p<0.01, ** p<0.05, * p<0.1 |  |  |  |  |  |  |  |  |
