# Supplementary material for: Proximity to small-scale inland and coastal fisheries is associated with improved income and food security
Source: Commun Earth Environ. 2022 Aug 3;3(1):174. doi: 10.1038/s43247-022-00496-5 (PMC9362682; doi:10.1038/s43247-022-00496-5)
Supplement: Supplementary file 3 — Supplementary Data 2 [file 43247_2022_496_MOESM3_ESM.docx]

**Table 2. Probit regression: estimated marginal effects at the mean distance to water bodies on the probability to be food insecure poor (households with poor food consumption score).**

| VARIABLES | All countries | | Malawi | | Tanzania | | Uganda | |
| --- | --- | --- | --- | --- | --- | --- | --- | --- |
|  | National | Rural | National | Rural | National | Rural | National | Rural |
| Distance to nearest water body (km) | 0.00032*** | 0.00074*** | 0.00012*** | 0.00043*** | 0.00046*** | 0.00054*** | 0.00045*** | 0.00061*** |
|  | (0.00000) | (0.00000) | (0.00001) | (0.00001) | (0.00000) | (0.00001) | (0.00001) | (0.00001) |
| Distance to nearest water body (km) of households unable to reach food markets | 0.00229*** | 0.00248*** | 0.00802*** | 0.00737*** | -0.00002 | -0.00034*** | 0.00179*** | 0.00152*** |
|  | (0.00002) | (0.00002) | (0.00018) | (0.00018) | (0.00011) | (0.00011) | (0.00001) | (0.00002) |
| Distance to nearest agricultural market (km) |  |  | 0.00231*** | -0.00023*** |  |  | 0.00025*** | -0.00002** |
|  |  |  | (0.00002) | (0.00002) |  |  | (0.00001) | (0.00001) |
| Neither fishing nor agriculture HHs, close to water bodies | 0.02227*** | -0.00054 | 0.14017*** | 0.01334*** | -0.00379*** | -0.10137*** | -0.02204*** | 0.02721*** |
|  | (0.00058) | (0.00113) | (0.00241) | (0.00287) | (0.00061) | (0.00110) | (0.00072) | (0.00136) |
| Fishing HHs, close to water bodies | 0.00361*** | 0.09029*** | -0.12426*** | -0.21099*** | -0.09952*** | -0.07870*** | 0.16763*** | 0.19475*** |
|  | (0.00136) | (0.00182) | (0.00528) | (0.00593) | (0.00123) | (0.00187) | (0.00388) | (0.00405) |
| Agriculture HHs, close to water bodies | -0.04130*** | -0.06489*** | -0.01278*** | -0.02707*** | -0.05838*** | -0.09652*** | -0.01754*** | -0.01172*** |
|  | (0.00033) | (0.00040) | (0.00098) | (0.00099) | (0.00041) | (0.00047) | (0.00061) | (0.00065) |
| Households unable to reach food markets | -0.07961*** | -0.09077*** | -0.29026*** | -0.29660*** | -0.09543*** | -0.12966*** | -0.03239*** | -0.02402*** |
|  | (0.00075) | (0.00096) | (0.00662) | (0.00770) | (0.00349) | (0.00303) | (0.00062) | (0.00072) |
| Fishing households | -0.05019*** | -0.09841*** | 0.06895*** | -0.02215*** | -0.05397*** | -0.06445*** | -0.06684*** | -0.07005*** |
|  | (0.00071) | (0.00096) | (0.00208) | (0.00234) | (0.00069) | (0.00091) | (0.00208) | (0.00232) |
| Agriculture households | 0.04790*** | 0.00657*** | 0.18420*** | 0.04203*** | 0.07826*** | 0.07137*** | -0.07963*** | -0.09065*** |
|  | (0.00029) | (0.00054) | (0.00085) | (0.00126) | (0.00034) | (0.00061) | (0.00056) | (0.00080) |
| Household consumed fish (past 7 days) | -0.21989*** | -0.20811*** | -0.26947*** | -0.23625*** | -0.20531*** | -0.17268*** | -0.15220*** | -0.13672*** |
|  | (0.00025) | (0.00030) | (0.00059) | (0.00057) | (0.00031) | (0.00038) | (0.00029) | (0.00033) |
| Household size | -0.01647*** | -0.01794*** | 0.00150*** | 0.00285*** | -0.01905*** | -0.01719*** | -0.00186*** | 0.00009 |
|  | (0.00004) | (0.00005) | (0.00017) | (0.00017) | (0.00006) | (0.00007) | (0.00005) | (0.00006) |
| Ratio employed household member over not employed | -0.04300*** | -0.05893*** | 0.00624*** | -0.01713*** | -0.10211*** | -0.13422*** | 0.10247*** | 0.06795*** |
|  | (0.00037) | (0.00048) | (0.00097) | (0.00098) | (0.00044) | (0.00059) | (0.00060) | (0.00069) |
| Age of the household head | 0.00029*** | 0.00261*** | -0.00727*** | -0.00567*** | 0.00354*** | 0.00980*** | -0.00688*** | -0.00627*** |
|  | (0.00004) | (0.00005) | (0.00010) | (0.00011) | (0.00005) | (0.00006) | (0.00006) | (0.00007) |
| Age of the household head, quadratic | 0.00001*** | -0.00001*** | 0.00008*** | 0.00006*** | -0.00003*** | -0.00008*** | 0.00008*** | 0.00007*** |
|  | (0.00000) | (0.00000) | (0.00000) | (0.00000) | (0.00000) | (0.00000) | (0.00000) | (0.00000) |
| Sex of the head of the household | -0.03137*** | -0.02588*** | -0.01656*** | -0.01281*** | -0.04320*** | -0.02924*** | 0.01471*** | 0.00775*** |
|  | (0.00024) | (0.00031) | (0.00065) | (0.00066) | (0.00030) | (0.00039) | (0.00034) | (0.00040) |
| Education of the head of the household - Primary | -0.07653*** | -0.07210*** | -0.13617*** | -0.11926*** | -0.08226*** | -0.06743*** | 0.00678*** | 0.00634*** |
|  | (0.00027) | (0.00033) | (0.00072) | (0.00079) | (0.00035) | (0.00041) | (0.00038) | (0.00042) |
| Education of the head of the household - Secondary | -0.15468*** | -0.17613*** | -0.30062*** | -0.25736*** | -0.15730*** | -0.13054*** | 0.02853*** | 0.01761*** |
|  | (0.00033) | (0.00045) | (0.00103) | (0.00144) | (0.00041) | (0.00063) | (0.00056) | (0.00068) |
| Wealth index | -0.00196*** | -0.00303*** | -0.05855*** | -0.05456*** |  | -0.00345*** | -0.07674*** | -0.10310*** |
|  | (0.00000) | (0.00001) | (0.00016) | (0.00017) |  | (0.00001) | (0.00019) | (0.00026) |
| Baseline: average probability to be food insecure | 0.270 | 0.315 | 0.549 | 0.633 | 0.210 | 0.248 | 0.174 | 0.177 |
| Average distance to water bodies, in Km. | 33.1 | 36.0 | 37.0 | 37.1 | 33.8 | 38.0 | 28.5 | 31.2 |
| Observations | 18,623 | 14,283 | 12,444 | 10,174 | 3,344 | 1,971 | 2,822 | 2,125 |
| Country/district FE | Yes | Yes | Yes | Yes | Yes | Yes | Yes | Yes |
| r2 | 0.1872 | 0.202 | 0.1829 | 0.1155 | 0.1188 | 0.1449 | 0.1781 | 0.1823 |
| Standard errors in parentheses *** p<0.01, ** p<0.05, * p<0.1 |  |  |  |  |  |  |  |  |
