# Supplementary material for: Proximity to small-scale inland and coastal fisheries is associated with improved income and food security
Source: Commun Earth Environ. 2022 Aug 3;3(1):174. doi: 10.1038/s43247-022-00496-5 (PMC9362682; doi:10.1038/s43247-022-00496-5)
Supplement: Supplementary file 6 — Description of Additional Supplementary Files [file 43247_2022_496_MOESM6_ESM.pdf]

Description of Additional Supplementary Files

File Name: Supplementary Data 1

Description: Probit regression: estimated marginal effects at the mean distance to water bodies on the probability to be income poor (living below the national poverty line)

File Name: Supplementary Data 2

Description: Probit regression: estimated marginal effects at the mean distance to water bodies on the probability to be food insecure poor (households with poor food consumption score).
